# T-Shaped Palladium and Platinum {MNO}^10^ Nitrosyl Complexes

**DOI:** 10.1021/acs.inorgchem.3c03434

**Published:** 2024-01-11

**Authors:** Matthew J. G. Sinclair, Nil Roig, Matthew R. Gyton, Nikolaos Tsoureas, F. Geoffrey N. Cloke, Mercedes Alonso, Adrian B. Chaplin

**Affiliations:** † Department of Chemistry, University of Warwick, Gibbet Hill Road, Coventry CV4 7AL, U.K.; ‡ Eenheid Algemene Chemie (ALGC), Vrije Universiteit Brussel (VUB), 1050 Brussels, Belgium; ∥ Department of Chemistry, University of Sussex, Falmer, Brighton BN1 9QR, U.K.

## Abstract

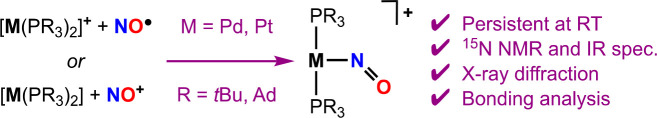

The synthesis and characterization of a homologous series of T-shaped {MNO}^10^ nitrosyl complexes of the form [M(PR_3_)_2_(NO)]^+^ (M = Pd, Pt; R = *t*Bu, Ad) are reported. These diamagnetic nitrosyls are obtained from monovalent or zerovalent precursors by treatment with NO and NO^+^, respectively, and are notable for distinctly bent M–NO angles of ∼123° in the solid state. Adoption of this coordination mode in solution is also supported by the analysis of isotopically enriched samples by ^15^N NMR spectroscopy. Effective oxidation states of M^0^/NO^+^ are calculated, and metal–nitrosyl bonding has been interrogated using DFT-based energy decomposition analysis techniques. While a linear nitrosyl coordination mode was found to be electronically preferred, the M–NO and P–M–P angles are inversely correlated to the extent that binding in this manner is prevented by steric repulsion between the bulky ancillary phosphine ligands.

## Abstract 2

The synthesis of mononuclear palladium and platinum {MNO}^10^ nitrosyl complexes of the form [M(PR_3_)_2_(NO)]^+^ (M = Pd, R = *t*Bu, Ad; Pt, R = *t*Bu, Ad) is reported. Experimental evidence indicates adoption of a bent M−NO coordination mode, and the underlying electronic features have been scrutinized using a DFT-based energy decomposition analysis.

Transition metal nitrosyl complexes are an important and widely established class of coordination compound,^1^ with the propensity for delocalization through the M–NO linkage embodied in the Enemark–Feltham classification scheme {MNO}^*n*^, where *n* is the sum of the metal *d*-electron count and the number of nitrosyl π* electrons.^2^ Although the biological importance of NO continues to motivate investigation of first row late transition metal nitrosyl complexes, the chemistry of second and third row congeners remains underexplored.^1,3^ This is somewhat surprising, given noble-metal-based materials play a key role in technologies for the abatement of environmentally damaging NO_*x*_ emissions, e.g., catalytic converters and industrial scale catalytic reduction processes.^4^ For instance, while mononuclear {NiNO}^10^ complexes are well established in the literature,^5^ palladium and platinum analogues are scarce and poorly defined, with [CpM(NO)] (M = Pd, Pt) reported by Fischer in the early 1960s the most notable examples.^6^ Crystallographically characterized palladium and platinum nitrosyls are limited to a handful of polynuclear and {MNO}^8^ systems.^7^ We herein report on the synthesis, isolation, and characterization of a homologous series of discrete {MNO}^10^ nitrosyl complexes of the form [M(PR_3_)_2_(NO)][BAr^F^_4_] (M = Pd, R = *t*Bu **1**, Ad **2**; Pt, R = *t*Bu **3**, Ad **4**; Ar^F^ = 3,5-(CF_3_)_2_C_6_H_3_; Figure 1), which address this knowledge gap.

**Figure 1:**
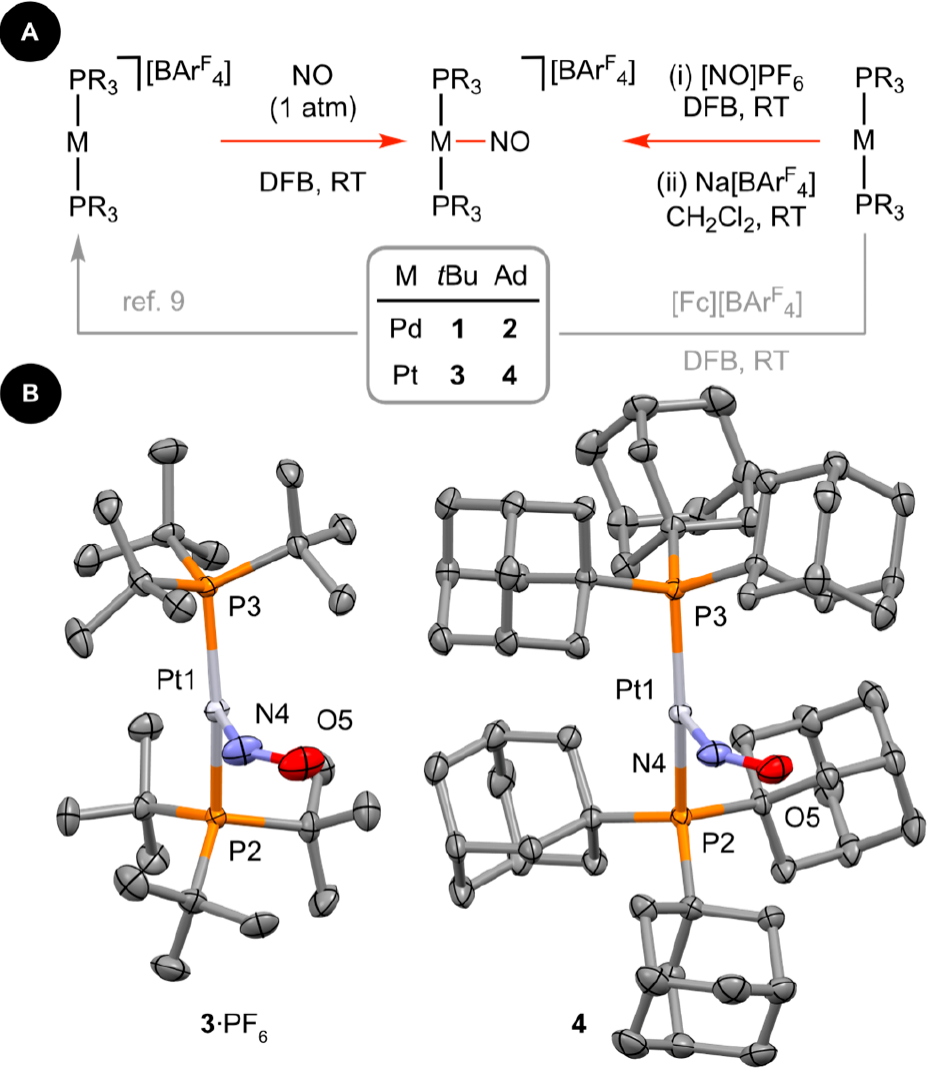
(A) Preparation of palladium and platinum {MNO}^10^ nitrosyl complexes **1**–**4**. (B) Solid-state structures of **3**·PF_6_ and **4** with thermal ellipsoids set at 50% probability. H atoms, counterions, and solvent molecules are omitted for clarity. The palladium complexes **1**·PF_6_ and **2** are isomorphous.

As part of work in our group investigating the chemistry of palladium(I) and platinum(I) metalloradicals,^8^ we have recently prepared the set of well-defined and persistent *d*^9^-metalloradicals [M(PR_3_)_2_][BAr^F^_4_] (M = Pd, Pt; R = *t*Bu, Ad) by one-electron oxidation of the corresponding zerovalent precursors [M(PR_3_)_2_] using [Fc][BAr^F^_4_] (Fc = FeCp_2_) in 1,2-difluorobenzene (DFB).^9^ Inspired by literature precedents for the addition of NO to second and third row late transition metal metalloradicals,^10,11^ we set about examining reactions of [M(PR_3_)_2_][BAr^F^_4_] with NO in DFB (Figure 1). Rapid reactions resulting in quantitative spectroscopic formation of diamagnetic {MNO}^10^ nitrosyl derivatives **1**–**4** were observed in all cases upon addition of NO at RT (M = Pd, dark red; Pt, dark green). The products are persistent in solution, stable to vacuum, and were subsequently isolated in >70% yield and extensively characterized (Figure 1, Table 1). Recognizing that nitrosonium is a stronger one-electron oxidant than ferrocenium,^12^ reactions of [M(PR_3_)_2_] (M = Pd, Pt; R = *t*Bu, Ad) with [NO]PF_6_ in DFB were also examined and found to be a useful alternative route to **1**–**4**, following salt metathesis of isolated [M(PR_3_)_2_(NO)]PF_6_ with Na[BAr^F^_4_] in CH_2_Cl_2_ at RT (41–75% isolated yield over two steps). In the case of tri-*tert*-butylphosphine-ligated nitrosyls **1** and **3**, the intermediate PF_6_^–^ salts **1**·PF_6_ and **3**·PF_6_ proved to be more amenable to analysis in the solid state by single-crystal X-ray diffraction than their [BAr^F^_4_]^−^ counterparts.

**Table 1:** Characterisation and Computational Data for [M(PR_3_)_2_(NO)]^+^**1**–**4**^a^

	**1**	**2**	**3**	**4**
M	Pd	Pd	Pt	Pt
R	*t*Bu	Ad	*t*Bu	Ad
M–NO/Å	1.907(2)^b^	1.900(2)	1.941(6)^b^	1.921(3)
N–O/Å	1.151(4)^b^	1.156(3)	1.157(11)^b^	1.166(4)
∠MNO/°	123.0(3)^b^	123.2(2)	122.1(7)^b^	123.2(2)
∠PMP/°	161.02(2)^b^	159.88(2)	162.60(6)^b^	161.65(2)
ν(NO)/cm^–1^	1712	1686	1659	1631
δ_31P_ (^1^*J*_PtP_)	85.4	72.6	80.3 (3983 Hz)	63.9 (3915 Hz)
δ_15N_ (^2^*J*_PN_)	855.3 (2 Hz)	862.6 (−)^c^	791.3 (3 Hz)	797.0 (2 Hz)
EOS (*R*%)^d^	Pd^0^/NO^+^ (76.9)	Pd^0^/NO^+^ (76.6)	Pt^0^/NO^+^ (68.1)	Pt^0^/NO^+^ (67.3)
Δ*E*_int_	–130.7	–139.6	–139.8	–149.4
Δ*E*_Pauli_	+206.7	+210.3	+253.4	+265.3
Δ*V*_elstat_	–114.2	–117.4	–135.5	–142.1
Δ*E*_orb_	–223.2	–232.5	–257.7	–272.6
*ΔE*_σ_ (%)	–156.6 (64.4)	–158.4 (62.7)	–177.1 (65.1)	–182.1 (63.5)
Δ*E*_π_ (%)	–50.2 (20.6)	–53.3 (21.1)	–51.6 (19.0)	–56.2 (19.6)
Δ*E*_rest_ (%)	–36.6 (15.0)	–40.7 (16.2)	–43.4 (15.9)	–48.3 (16.9)
Δ*E*_prep_	+22.5	+22.3	+26.3	+26.9
Δ*E*_bind_ (−*D*_*e*_)	–108.2	–117.3	–113.5	–122.5

a Experimental data for the [BAr^F^_4_]^−^salts unless otherwise stated, NMR data recorded in DFB and IR data collected in the solid-state using the ATR method. Energy decomposition based on {M(PR_3_)_2_}/NO^+^ fragmentation and performed at the ZORA-M06/TZ2P level of theory. Computed energies in kcal·mol^–1^.

b Crystallographic data for the PF_6_^–^salts.

c No coupling resolved.

d *R*% is the reliability index, which gauges how well the associated effective fragment orbitals model the electronic structure.

Complexes **1**–**4** adopt distorted T-shaped geometries (P–M–P ∼ 161°) in the solid state with M–NO angles of ∼123° and are correspondingly classified as bent nitrosyls. This assignment is also borne out by analysis of isotopically enriched samples in DFB solution by NMR spectroscopy, where characteristically downfield ^15^N resonances were located at δ 855.3, **1**; 862.6, **2**; 791.3, **3**; and 797.0, **4**.^13^ Coordination of NO was also confirmed by ATR-IR spectroscopy, ESI-MS, and combustion analysis. The ν(NO) bands are not structurally diagnostic in this case but are significantly red-shifted relative to free NO (1875 cm^–1^), decreasing in the order **1** (1712 cm^–1^) > **2** (1686 cm^–1^) > **3** (1659 cm^–1^) > **4** (1631 cm^–1^) viz. M = Pd > Pt and R = *t*Bu > Ad. For context, cyclopentadienyl complexes [CpM(NO)] are characterized by ν(NO) bands at 1789 cm^–1^ (M = Pd) and 1739 cm^–1^ (M = Pt).^6^ There is no spectroscopic or crystallographic evidence for the adoption of supporting agostic interactions, typically expected for three-coordinate *d*^8^-complexes (M–C > 3.4 Å in all cases).^14^ Previously reported three-coordinate {MNO}^10^ nitrosyls are limited to trigonal planar nickel complexes which invariably adopt linear M–NO geometries, exemplified by [Ni(nacnac)(NO)] (nacnac = aryl substituted β-diketiminate, ν(NO) = 1784–1825 cm^–1^) and [Ni(dtbpe)(NO)]^+^ (dtbpe = 1,2-bis(di-*tert*-butylphosphino)ethane, ν(NO) = 1836 cm^–1^).^5^ Examples of distinctly bent {MNO}^10^ nitrosyls can, however, be found in the literature with higher coordination numbers for M = Ni and Cu.^15^

To help understand the electronic structure of these unprecedented {MNO}^10^ nitrosyl complexes, **1**–**4** were examined computationally (Table 1). Structures were optimized at the M06/def2SVP level of theory and provide geometric parameters in very good agreement with experiment.^16^ Analysis using Salvador’s Effective Oxidation State (EOS) method indicates that, while the M–NO bonds have a high degree of covalency, the electron pair goes to the metal when assigning formal oxidation states in these complexes (M^0^/NO^+^).^17,18^ Subsequent energy decomposition analysis was carried out using the extended transition state (ETS) method in combination with natural orbitals for chemical valence (NOCV) theory at the ZORA-M06/TZ2P level of theory.^19,20^ The calculated bond dissociation energies (*D*_e_/kcal·mol^–1^) increase in the order **1** (108.2) < **3** (113.5) < **2** (117.3) < **4** (122.5), viz. M = Pd < Pt and R = *t*Bu < Ad. Coordination of the nitrosyl ligand is characterized by a large degree of Pauli repulsion, in line with the destabilization expected for interaction between the nitrogen lone pair of a bent nitrosyl and a low-valent late transition metal. This repulsive contribution is overcome by stabilizing charge-transfer interactions from the metal *d*-orbitals into the nitrosyl π* orbitals (Figure 2), and there is good agreement between the trend in Δ*E*_orb_ and ν(NO) observed experimentally. The largest component is σ-bonding in character (62.7–65.1% Δ*E*_orb_) and is supplemented by in-plane π-bonding (19.0–21.1% Δ*E*_orb_).

**Figure 2:**
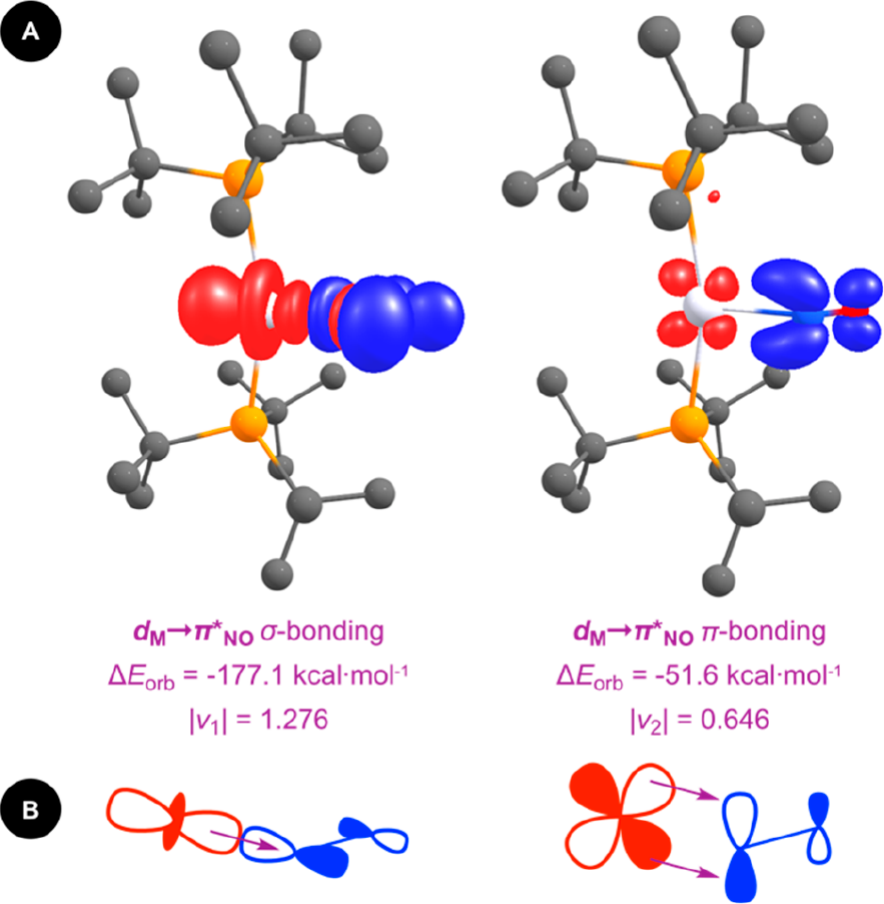
(A) Leading ETS-NOCV deformation densities associated with {Pt(P*t*Bu_3_)_2_}/NO^+^ fragmentation of **3**, charge flow from red to blue. Associated interaction energies and eigenvalues. (B) Schematic depiction of the orbital interactions.

The capacity to adopt a linear nitrosyl coordination mode has been investigated *in silico* for **1** and **3**, through relaxed potential energy scans of the M–NO angle from the equilibrium values up to ∼176°. This distortion correlates with compression of the P–M–P angle (∼30° over the scan) and incurs a significant energetic penalty (13.5 kcal·mol^–1^ for **1** and 16.4 kcal·mol^–1^ for **3**; Figure 3). The formal oxidation states are unchanged, with EOSs of M^0^/NO^+^ calculated for the pseudo linear isomers of **1** and **3** with high reliability scores. These findings reinforce the electronic link between trigonal planar metal geometry and linear nitrosyl coordination modes for three-coordinate {MNO}^10^ complexes, which is evident from the known nickel precedents and can be reconciled by qualitative analysis of the metal frontier molecular orbitals using a Walsh diagram for a *d*^10^-ML_2_ fragment (see the Supporting Information).^5,21^ Indeed, examination of the profiles using the activation strain model (ASM) indicates that, while the linear coordination mode gives rise to more energetically favorable metal–nitrosyl interactions (−1.9 kcal·mol^–1^ for **1**; –10.5 kcal·mol^–1^ for **3**), the strain energy associated with perturbation of the {M(P*t*Bu_3_)_2_} fragments from linearity is prohibitively large (+15.4 kcal·mol^–1^ for **1**, +26.9 kcal·mol^–1^ for **3**). Complexes **1**–**4** can, therefore, be considered “frustrated linear nitrosyls”.

**Figure 3:**
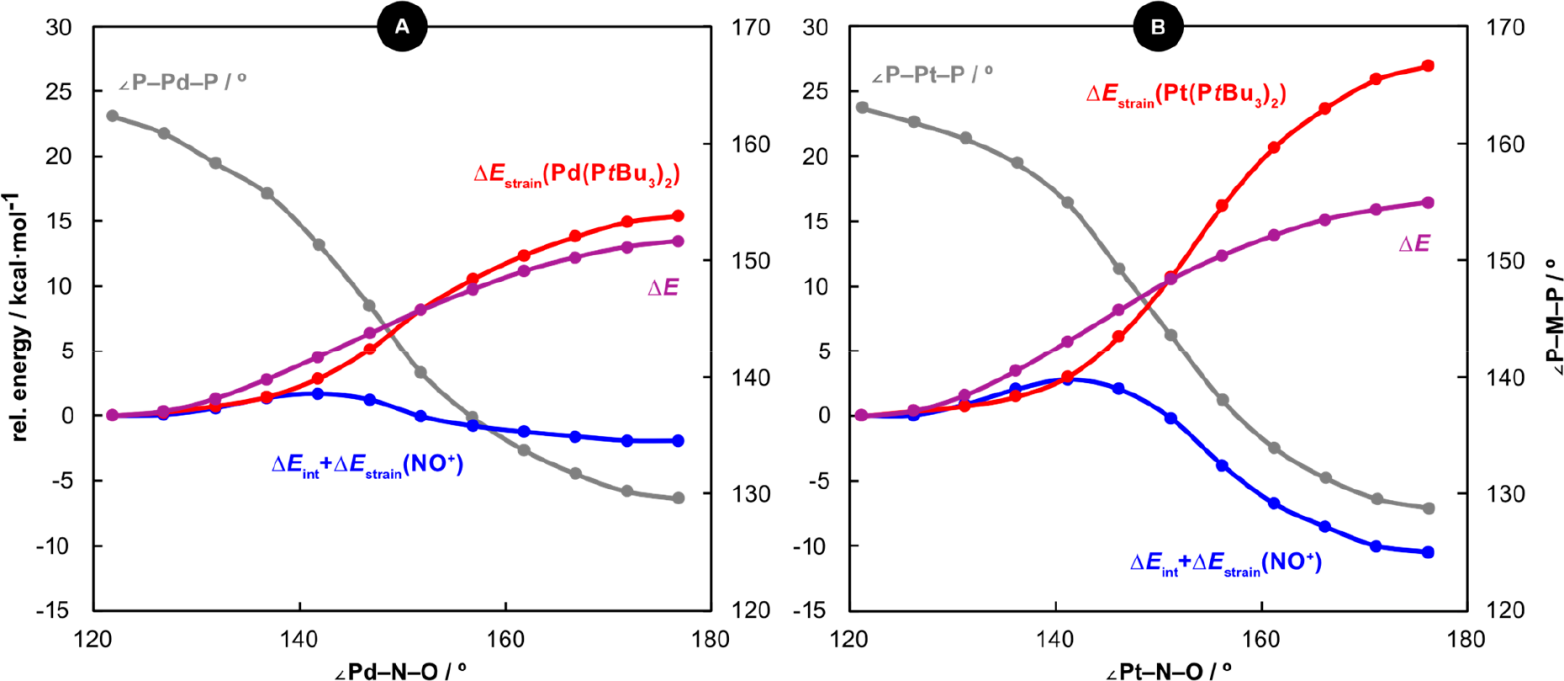
Relaxed potential energy scan for variation of the ∠M–N–O angles in **1** (A) and **3** (B); change in electronic energy (Δ*E*) in purple and ∠P–M–P angle in gray. The potential energy surface is further deconvoluted by ASM analysis of the {M(P*t*Bu_3_)_2_}/NO^+^ fragmentation (red and blue).

In summary, we report the synthesis and comprehensive characterization of four discrete palladium and platinum {MNO}^10^ nitrosyl complexes. In contrast to isoelectronic nickel systems, adoption of a distinctly bent nitrosyl coordination mode is evident in the solid state by X-ray diffraction (M–NO ∼ 123°) and in solution by characteristically downfield ^15^N NMR resonances. Calculations indicate that the use of bulky ancillary phosphine ligands is decisive in this regard and highlights, more generally, the importance of ligand sterics in the activation of nitric oxide by late transition metal complexes.

## Safety Statement

**Caution!***Nitric oxide is a condensable gas that reacts with air, is highly toxic and is corrosive to the respiratory tract. It is also oxidizing and may react violently with organic compounds. This gas should be handled with extreme care in a well-ventilated fume hood using vacuum line techniques. Manipulations involving nitric oxide were performed on the smallest practical scale as described in the*Supporting Information, *and a procedure for the preparation of***1***–***4***that does not require nitric oxide is described.*

## Supporting Information Available

NMR, IR and ESI-MS spectra of complexes; further computational details, data, and analysis (PDF)
Optimized geometries of species examined computationally (XYZ)

## Supplementary Material

ic3c03434_si_001.pdf

ic3c03434_si_002.xyz
