# Supplementary material for: T-Shaped Palladium and Platinum {MNO}10 Nitrosyl Complexes
Source: Inorg Chem. 2024 Jan 11;63(4):1709–13. doi: 10.1021/acs.inorgchem.3c03434 (PMC10828984; doi:10.1021/acs.inorgchem.3c03434)
Supplement: Supplementary file 1 — ic3c03434_si_001.pdf [file ic3c03434_si_001.pdf]

Supporting information for:

T-Shaped Palladium and Platinum $\{MNO\}^{10}$ Nitrosyl Complexes

Matthew J. G. Sinclair,[a] Nil Roig,[a,b] Matthew R. Gyton,[a] Nikolaos Tsoureas,[c,d]
F. Geoffrey N. Cloke,[c] Mercedes Alonso,[b] and Adrian B. Chaplin[a,*]

[a] *Department of Chemistry, University of Warwick, Gibbet Hill Road, Coventry CV4 7AL, UK.*
E-mail: a.b.chaplin@warwick.ac.uk
[b] *Eenheid Algemene Chemie (ALGC), Vrije Universiteit Brussel (VUB), 1050 Brussels, Belgium.*
[c] *Department of Chemistry, University of Sussex, Falmer, Brighton BN1 9QR, UK.*
[d] *Current address: Department of Chemistry, National and Kapodistrian University of Athens, Panepistimioupoli Zografou, 157 84 Athens, Greece.*

Table of contents

1. Experimental details 2
   1.1. General methods 2
   1.2. NMR scale reactions of $[M(PR_3)_2][BAr^F_4]$ with NO 2
   1.3. General procedures 3
   1.4. Preparation and characterisation of $[Pd(PtBu_3)_2(NO)][BAr^F_4]$ 3
   1.5. Preparation and characterisation of $[Pd(PtBu_3)_2(NO)][PF_6]$ 6
   1.6. Preparation and characterisation of $[Pd(PAd_3)_2(NO)][BAr^F_4]$ 8
   1.7. Preparation and characterisation of $[Pd(PAd_3)_2(NO)][PF_6]$ 11
   1.8. Preparation and characterisation of $[Pt(PtBu_3)_2(NO)][BAr^F_4]$ 13
   1.9. Preparation and characterisation of $[Pt(PtBu_3)_2(NO)][PF_6]$ 16
   1.10. Preparation and characterisation of $[Pt(PAd_3)_2(NO)][BAr^F_4]$ 18
   1.11. Preparation and characterisation of $[Pt(PAd_3)_2(NO)][PF_6]$ 21
2. Computational details 22
   2.1. General methods 22
   2.2. Effective oxidation state calculations 23
   2.3. Energy decomposition analysis 26
   2.4. Relaxed potential energy scans 28
   2.5. Activation strain model analysis 29
   2.6. Analysis of the ∠M–N–O rPESs 30
3. References 34

1. Experimental details

1.1. General methods

All manipulations were performed under an atmosphere of argon (1 atm) using Schlenk and glove box techniques unless otherwise stated. Glassware was oven dried at 150 °C overnight and flame-dried under vacuum prior to use. Molecular sieves were activated by heating at 300 °C *in vacuo* overnight. 1,2-Difluorobenzene (DFB) was pre-dried over Al_2O_3, distilled from calcium hydride and dried over two successive batches of 3 Å molecular sieves.[1] CD_2Cl_2 was freeze-pump-thaw degassed and dried over 3 Å molecular sieves. Anhydrous CH_2Cl_2 and hexane were purchased from Acros or Sigma-Aldrich, freeze-pump-thaw degassed and stored over 3 Å molecular sieves. [M(P*t*Bu$_3$)$_2$] (M = Pd, Pt) were purchased from Sigma-Aldrich or Strem Chemicals and recrystallised from hexane before use. [M(PAd)$_2$] (M = Pd, Pt) and [M(PR$_3$)$_2$][BAr$^F_4$] (Ar^F = 3,5-(CF$_3$)$_2$C$_6$H$_3$; M = Pd, Pt; R = *t*Bu, Ad) were prepared as previously described.[2] NO was purchased from BOC (Research Grade, N2.5) and used as received. ^{15}NO was synthesised from NOSO$_4$H and Hg according to a published procedure.[3] [NO]PF$_6$ was purchased from Alfa Aesar and used as received. Na[BAr$^F_4$] was prepared using a literature procedure.[4] NMR spectra were recorded on Bruker spectrometers under argon at 298 K unless otherwise stated. Chemical shifts are quoted in ppm and coupling constants in Hz. Virtual coupling constants are reported as the separation between the first and third lines.[5] NMR spectra in DFB were recorded using an internal capillary of C_6D_6.[1] ^{15}N NMR spectra are referenced to formamide in (CD$_3$)$_2$SO (90%, δ 112.0 relative to $^{15}NH_3$).[6] IR spectra were recorded on a Bruker Alpha Platinum ATR FT-IR spectrometer at room temperature under a dinitrogen atmosphere. High resolution (HR) ESI-MS analyses were recorded on Bruker Maxis Impact instrument. Microanalyses were performed at the London Metropolitan University by Stephen Boyer.

1.2. NMR scale reactions of [M(PR$_3$)$_2$][BAr$^F_4$] with NO

General procedure: DFB (0.5 mL) was vacuum distilled onto a sample of [M(PR$_3$)$_2$][BAr$^F_4$] (10 µmol) within a J. Young valve NMR tube, which was then thawed under NO (1 atm). After standing at room temperature for 5 min, the sample was freeze-pump-thaw degassed and placed under an atmosphere of argon. For each sample, analysis of the reaction *in situ* by ^{1}H and ^{31}P NMR spectroscopy indicated quantitative spectroscopic conversion into the corresponding nitrosyl derivative [M(PR$_3$)$_2$(NO)][BAr$^F_4$].

General procedure for analysis of [M(PR$_3$)$_2$(NO)][BAr$^F_4$] by ^{15}N NMR spectroscopy: A solution of [M(PR$_3$)$_2$][BAr$^F_4$] (10 µmol) in DFB (0.5 mL) within a J. Young valve NMR tube was freeze-pump-thaw degassed. Whilst the sample was frozen, ^{15}NO (*ca.* 100 µmol) was added using a Toepler pump (calibrated measuring volume 71.5 mL). The solution was thawed to room temperature and vigorously mixed for 10 min before volatiles were removed *in vacuo*. The resulting nitrosyl derivative was analysed by ^{15}N NMR spectroscopy in DFB (0.5 mL) under an atmosphere of argon without further purification.

1.3. General procedures

General procedure A: A solution of $[M(PR_3)_2][BAr^F_4]$ (20 mmol·L^{-1}) in DFB was freeze-pump-thaw degassed and placed under NO (1 atm). The reaction was stirred at room temperature for 15 min and volatiles were removed *in vacuo*. Recrystallisation from DFB/hexane at room temperature under an atmosphere of argon afforded the corresponding nitrosyl derivative $[M(PR_3)_2(NO)][BAr^F_4]$.

General procedure B: A suspension of $[M(PR_3)_2]$ (20 mmol·L^{-1}) and $[NO]PF_6$ (0.95 equiv.) in DFB was stirred at room temperature for 1 h. The solution was filtered, concentrated, and then layered with hexane to afford the corresponding nitrosyl derivative $[M(PR_3)_2(NO)][PF_6]$ as a crystalline solid upon diffusion at room temperature.

General procedure C: A suspension of $[M(PR_3)_2(NO)][PF_6]$ (10 mmol·L^{-1}) and $Na[BAr^F_4]$ (1.1 equiv.) in CH_2Cl_2 was stirred at room temperature for 1 h. The solution was filtered and the layered with hexane to afford the corresponding $[BAr^F_4]^-$ salt as a crystalline solid upon diffusion at room temperature.

1.4. Preparation and characterisation of [Pd(P*t*Bu$_3$)$_2$(NO)][BAr$^F_4$]

Following general procedure A using [Pd(P*t*Bu$_3$)$_2$][BAr$^F_4$] (27.4 mg, 19.9 µmol), the product was obtained as a dark red crystalline solid. Yield: 19.5 mg (13.9 µmol, 70%). Following general procedure C using [Pd(P*t*Bu$_3$)$_2$(NO)][PF$_6$] (48.9 mg, 72.6 µmol) and Na[BAr$^F_4$] (71.1 mg, 80.2 µmol), the product was obtained as a dark red crystalline solid. Yield: 86.8 mg (61.8 µmol, 85%).

^{1}H NMR (400 MHz, DFB): *δ* 8.11–8.16 (m, 8H, Ar^F), 7.50 (br, 4H, Ar^F), 1.24 (vt, J_{PH} = 13.1, 54H, *t*Bu).
^{1}H NMR (500 MHz, CD_2Cl_2): *δ* 7.71–7.75 (m, 8H, Ar^F), 7.56 (br, 4H, Ar^F), 1.47 (vt, J_{PH} = 13.0, 54H, *t*Bu).
^{13}C{^{1}H} NMR (126 MHz, CD_2Cl_2): *δ* 162.3 (q, $^1J_{CB}$ = 50, Ar^F), 135.4 (s, Ar^F), 129.5 (qq, $^2J_{FC}$ = 32, $^2J_{CB}$ = 3, Ar^F), 125.2 (q, $^1J_{FC}$ = 272, Ar^F), 118.0 (sept., $^3J_{FC}$ = 4, Ar^F), 40.3 (vt, J_{PC} = 6, *t*Bu{C}), 32.9 (s, *t*Bu{CH$_3$}).
^{31}P{^{1}H} NMR (162 MHz, DFB): *δ* 85.4 (s).
^{31}P{^{1}H} NMR (162 MHz, CD_2Cl_2): *δ* 85.6 (s).
^{15}N NMR (61 MHz, DFB, ^{15}NO labelled sample): *δ* 855.3 (t, $^2J_{PN}$ = 2).
IR (ATR): 1711 (NO) cm^{-1}.
HR ESI-MS (positive ion, 4 kV): 540.2723 ([*M*]$^+$, calcd 540.2720) *m/z*.
Anal. calcd for $C_{56}H_{66}BF_{24}NOP_2Pd$ (1404.29 g·mol^{-1}): C, 47.90; H, 4.74; N, 1.00. Found: C, 47.99; H, 4.48; N, 0.97.

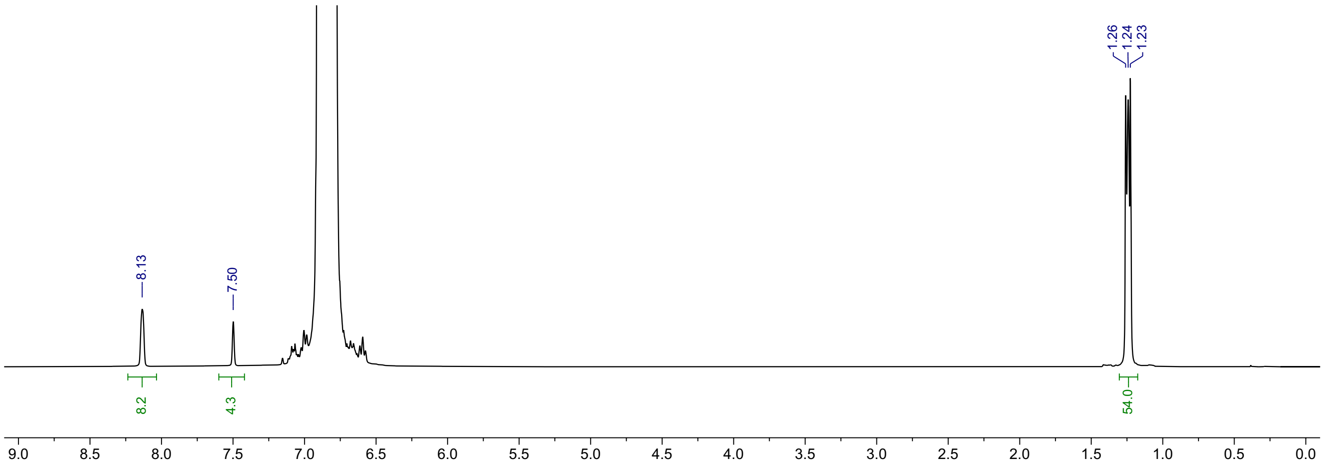

Figure S1. ^{1}H NMR spectrum of $[Pd(P\textit{t}Bu_3)_2(NO)][BAr^F{}_4]$ in DFB (400 MHz).

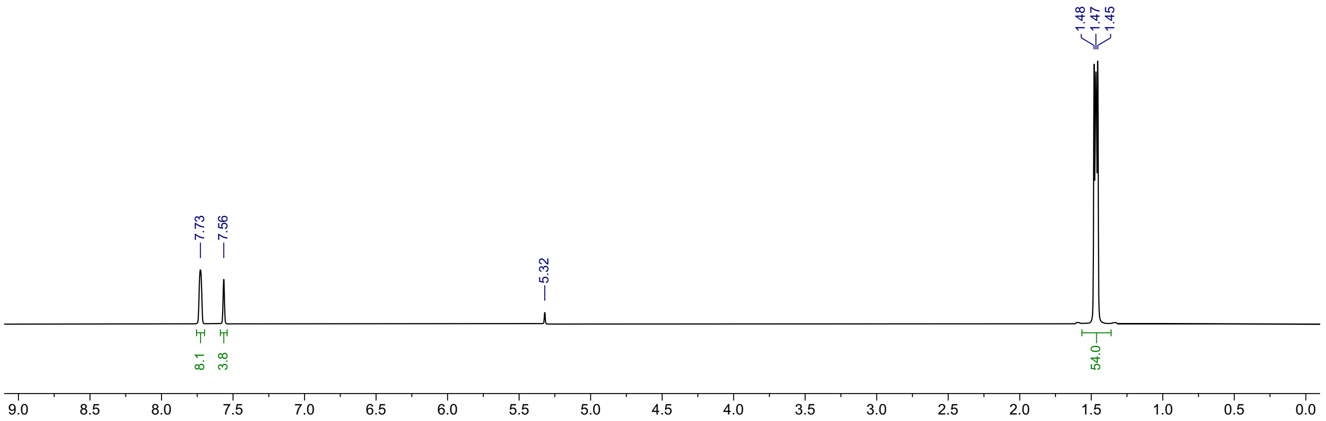

Figure S2. ^{1}H NMR spectrum of $[Pd(P\textit{t}Bu_3)_2(NO)][BAr^F{}_4]$ in CD_2Cl_2 (500 MHz).

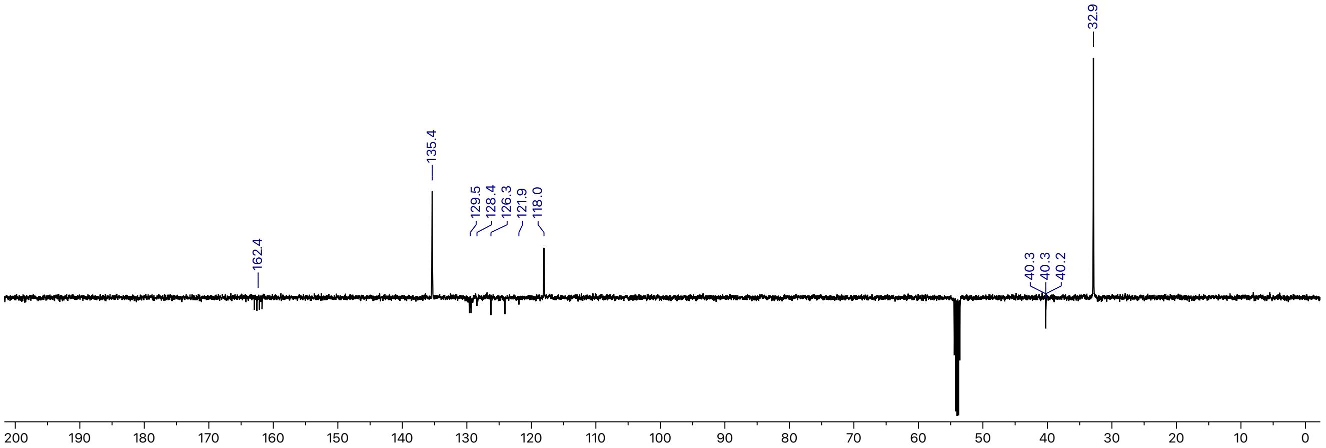

Figure S3. $^{13}C\{^{1}H\}$ APT NMR spectrum of $[Pd(P\textit{t}Bu_3)_2(NO)][BAr^F{}_4]$ in CD_2Cl_2 (126 MHz).

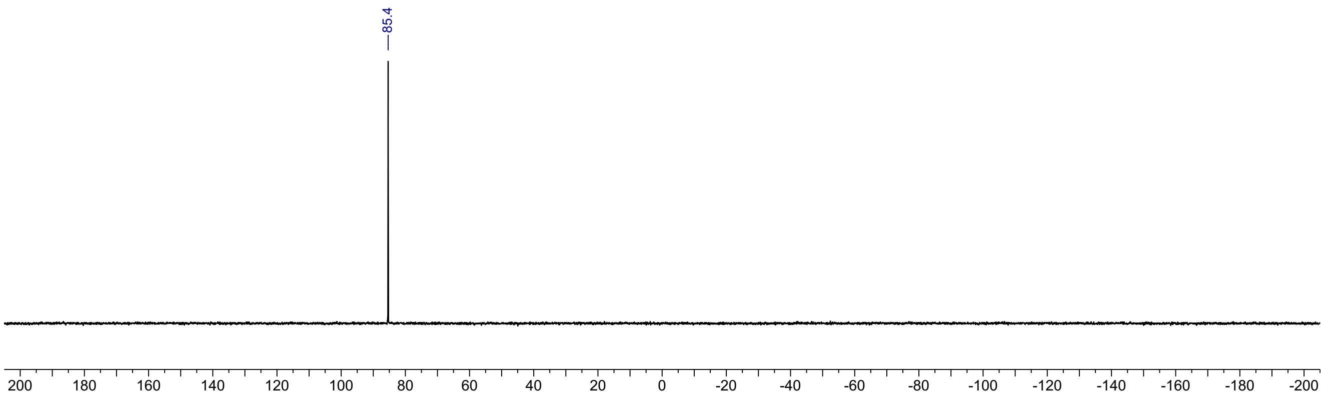

Figure S4. $^{31}P\{^{1}H\}$ NMR spectrum of $[Pd(PtBu_3)_2(NO)][BAr^F_4]$ in DFB (162 MHz).

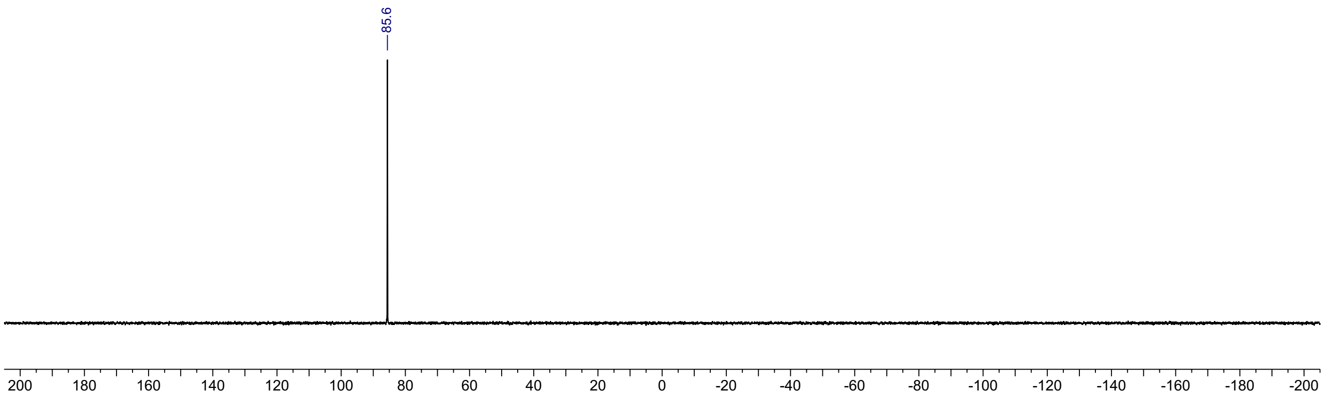

Figure S5. $^{31}P\{^{1}H\}$ NMR spectrum of $[Pd(PtBu_3)_2(NO)][BAr^F_4]$ in CD_2Cl_2 (162 MHz).

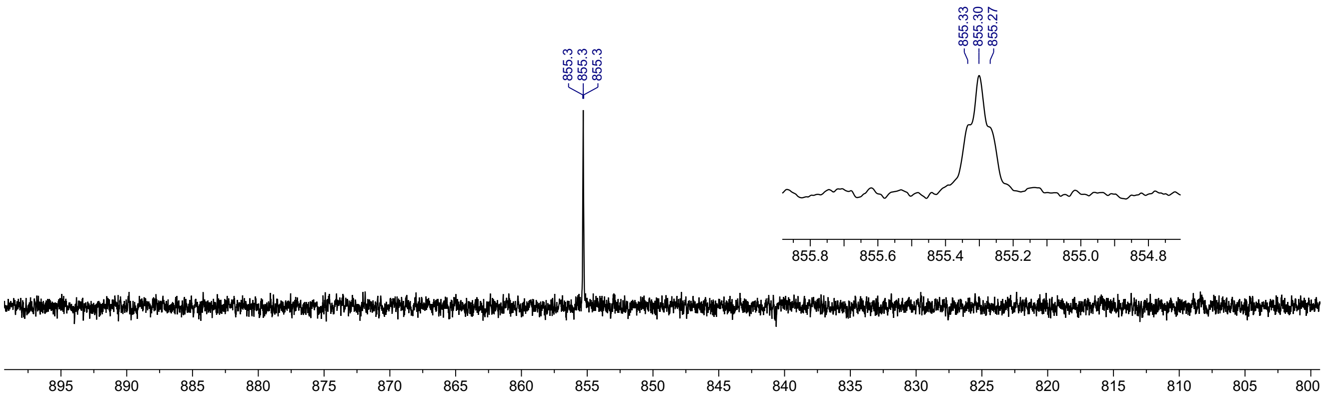

Figure S6. ^{15}N NMR spectrum of $[Pd(PtBu_3)_2(NO)][BAr^F_4]$ in DFB (61 MHz).

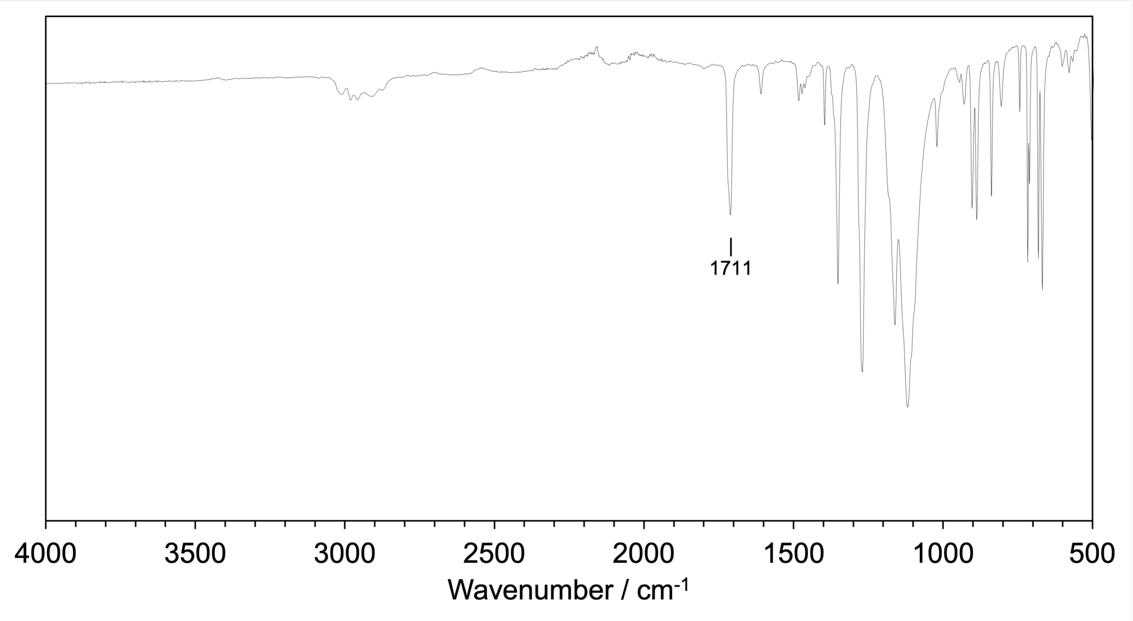

Figure S7. ATR-IR spectrum of $[Pd(PtBu_3)_2(NO)][BAr^F_4]$.

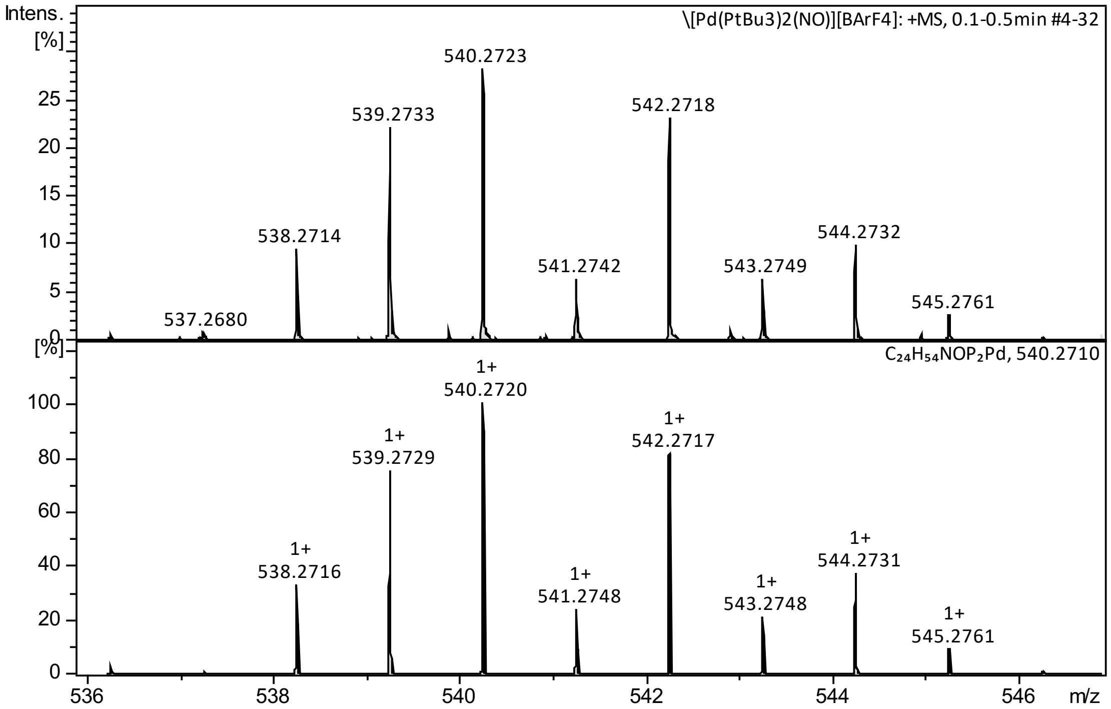

Figure S8. HR ESI-MS of $[Pd(PtBu_3)_2(NO)][BAr^F_4]$.

1.5. Preparation and characterisation of $[Pd(PtBu_3)_2(NO)][PF_6]$

Following general procedure B using $[Pd(PtBu_3)_2]$ (250 mg, 489 µmol) and $[NO]PF_6$ (81.1 mg, 464 µmol), the product was obtained as a dark red crystalline solid. Yield: 271 mg (395 µmol, 85%).

^{1}H NMR (400 MHz, DFB): *δ* 1.23 (vt, J_{PH} = 13.1, 54H, *t*Bu).

^{31}P{^{1}H} NMR (162 MHz, DFB): *δ* 85.3 (s, 2P, P*t*Bu₃), -143.2 (hept, $^1J_{PF}$ = 710, 1P, PF_6).

HR ESI-MS (positive ion, 4 kV): 540.2719 ($[M]^+$, calcd 540.2720) *m/z*.

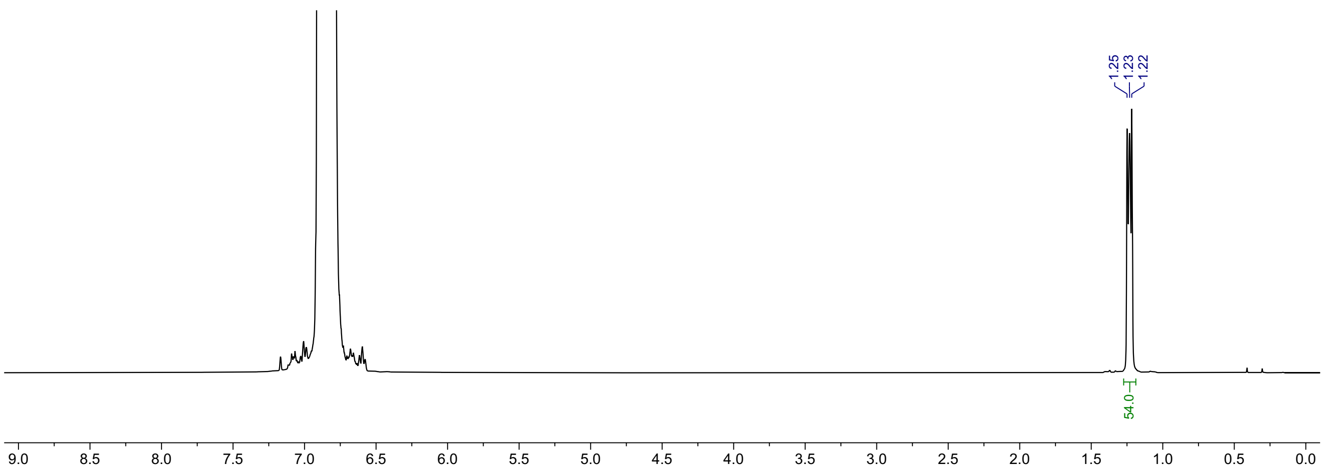

Figure S9. ^{1}H NMR spectrum of [Pd(P*t*Bu$_3$)$_2$(NO)][PF$_6$] in DFB (400 MHz).

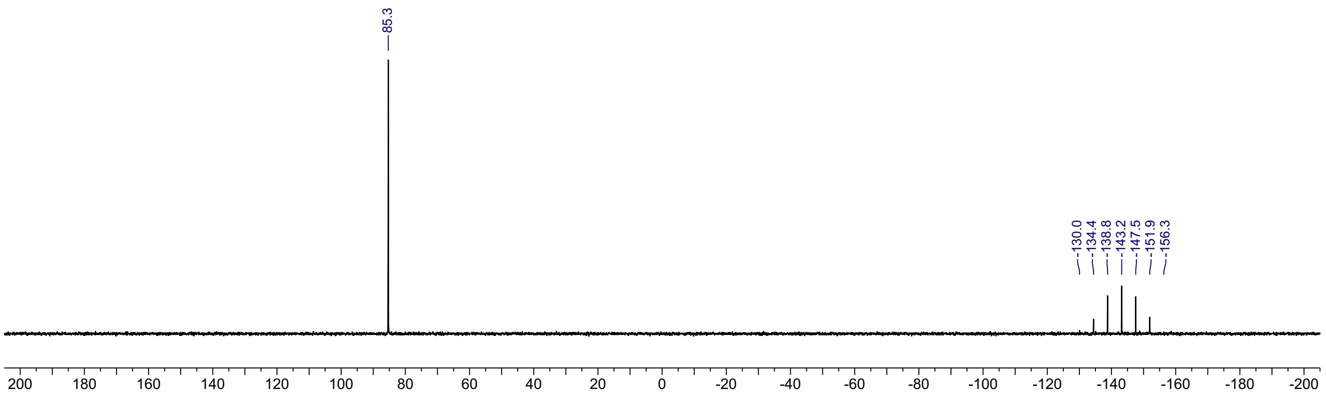

Figure S10. $^{31}P\{^{1}H\}$ NMR spectrum of [Pd(P*t*Bu$_3$)$_2$(NO)][PF$_6$] in DFB (162 MHz).

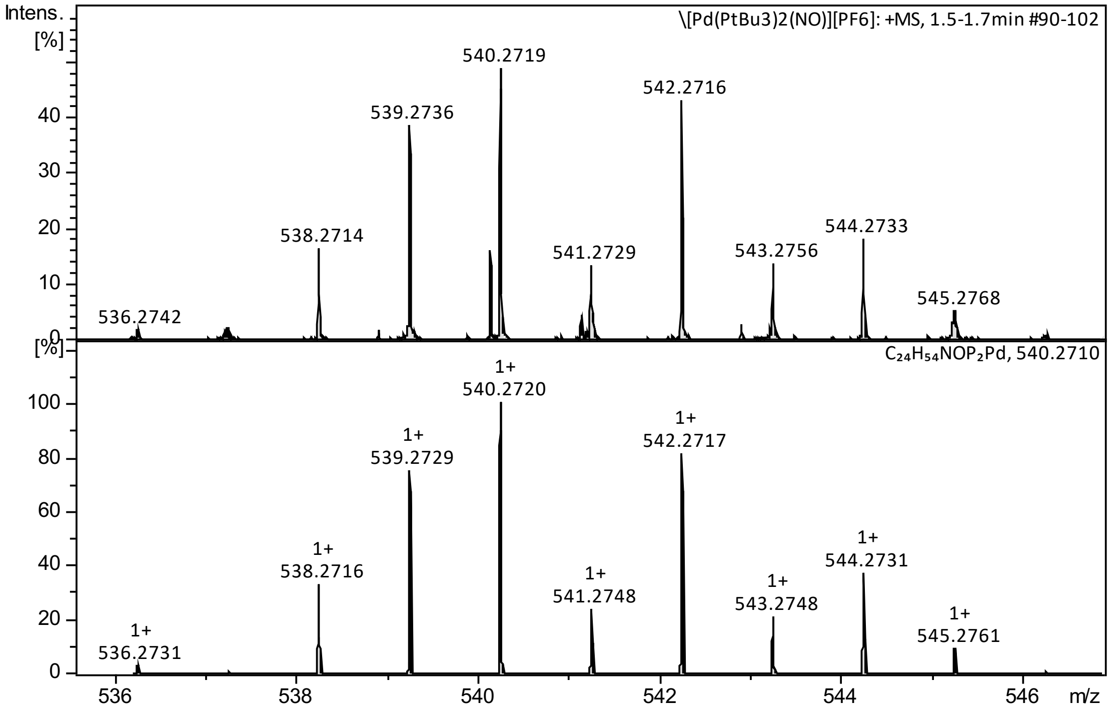

Figure S11. HR ESI-MS of [Pd(P*t*Bu$_3$)$_2$(NO)][PF$_6$].

1.6. Preparation and characterisation of $[Pd(PAd_3)_2(NO)][BAr^F_4]$

Following general procedure A using $[Pd(PAd_3)_2][BAr^F_4]$ (36.9 mg, 20.0 μmol), the product was obtained as a dark red crystalline solid. Yield: 37.1 mg (19.8 μmol, 99%). Following general procedure C using $[Pd(PAd_3)_2(NO)][PF_6]$ (6.5 mg, 5.6 μmol) and $Na[BAr^F_4]$ (6.0 mg, 6.8 μmol), the product was obtained as a dark red crystalline solid. Yield: 7.8 mg (4.2 μmol, 75%).

^{1}H NMR (400 MHz, DFB): δ 8.11–8.17 (m, 8H, Ar^F), 7.50 (br, 4H, Ar^F), 2.34 (br, 36H, Ad{2-CH_2}), 1.93 (s, 18H, Ad{3-CH}), 1.71 (d, $^2J_{HH}$ = 12.3, 18H, Ad{4-CH_2}), 1.64 (d, $^2J_{HH}$ = 12.3, 18H, Ad{4-CH_2}).
^{1}H NMR (600 MHz, CD_2Cl_2): δ 7.71–7.75 (m, 8H, Ar^F), 7.56 (br, 4H, Ar^F), 2.37 (br, 36H, Ad{2-CH_2}), 2.07 (s, 18H, Ad{3-CH}), 1.79 (unresolved AB resonances, 36H, 2×Ad{4-CH_2}).
^{13}C{^{1}H} NMR (151 MHz, CD_2Cl_2): δ 162.3 (q, $^1J_{CB}$ = 50, Ar^F), 135.4 (s, Ar^F), 129.4 (qq, $^2J_{FC}$ = 32, $^2J_{CB}$ = 3, Ar^F), 125.2 (q, $^1J_{FC}$ = 272, Ar^F), 118.0 (sept., $^3J_{FC}$ = 4, Ar^F), 48.5 (br, Ad{1-C}), 44.1 (observed in HSQC and HMBC only, Ad{2-CH_2}), 36.8 (s, Ad{4-CH_2}), 29.9 (vt, J_{PC} = 7, Ad{3-CH}).
^{31}P{^{1}H} NMR (162 MHz, DFB): δ 72.6 (s).
^{31}P{^{1}H} NMR (162 MHz, CD_2Cl_2): δ 72.8 (s).
^{15}N NMR (61 MHz, DFB, ^{15}NO labelled sample): δ 862.6 (br).
IR (ATR): 1686 (NO) cm^{-1}.
HR ESI-MS (positive ion, 4 kV): 1008.5544 ($[M]^+$, calcd 1008.5547) *m/z*.
Anal. calcd for $C_{92}H_{102}BF_{24}NOP_2Pd$ (1872.97 g·mol^{-1}): C, 59.00; H, 5.49; N, 0.75. Found: C, 58.86; H, 5.34; N, 0.68.

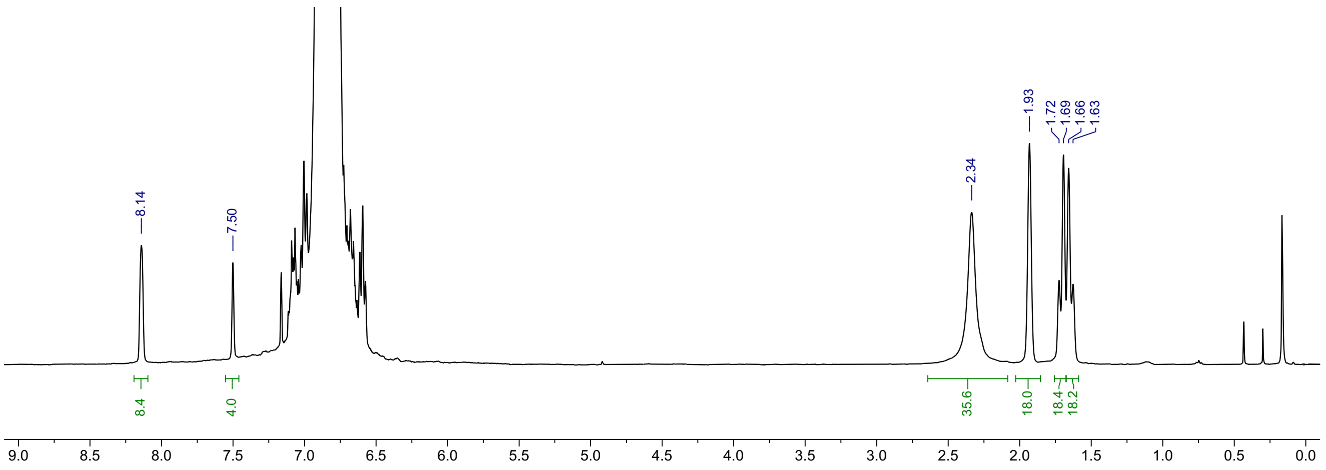

Figure S12. ^{1}H NMR spectrum of $[Pd(PAd_3)_2(NO)][BAr^F_4]$ in DFB (400 MHz).

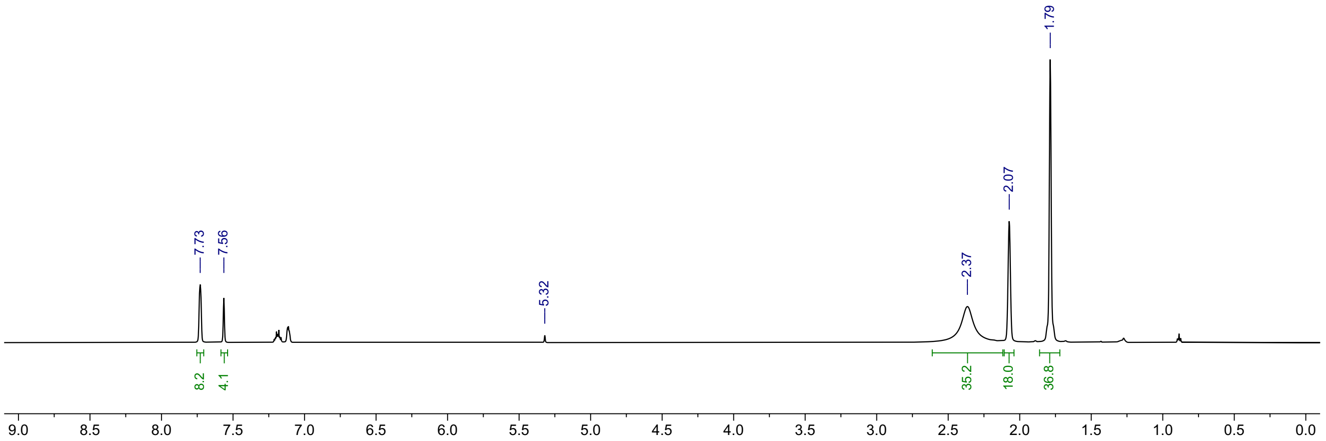

Figure S13. ^{1}H NMR spectrum of $[Pd(PAd_3)_2(NO)][BAr^F_4]$ in CD_2Cl_2 (600 MHz). Sample contains residual DFB from recrystallisation.

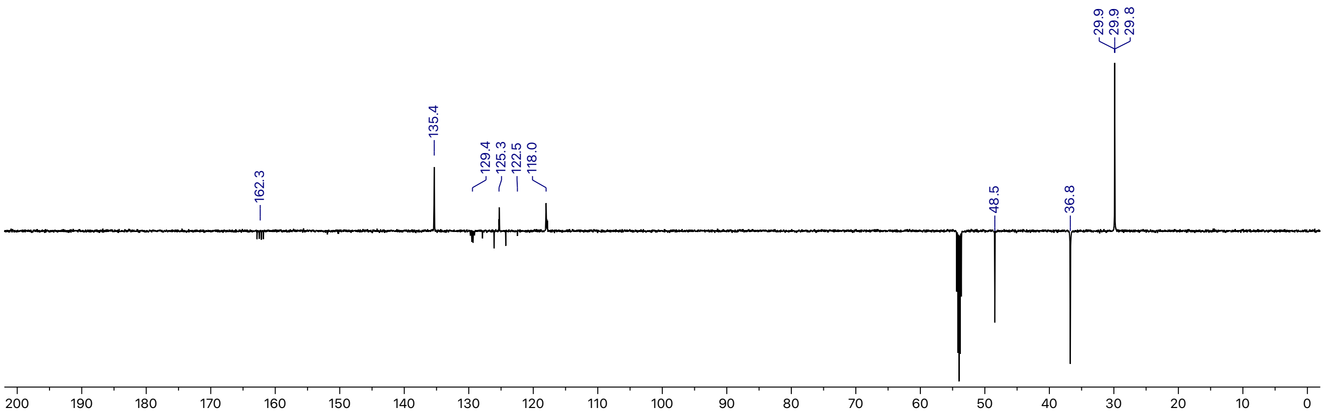

Figure S14. $^{13}C\{^{1}H\}$ APT NMR spectrum of $[Pd(PAd_3)_2(NO)][BAr^F_4]$ in CD_2Cl_2 (151 MHz). Sample contains residual DFB from recrystallisation.

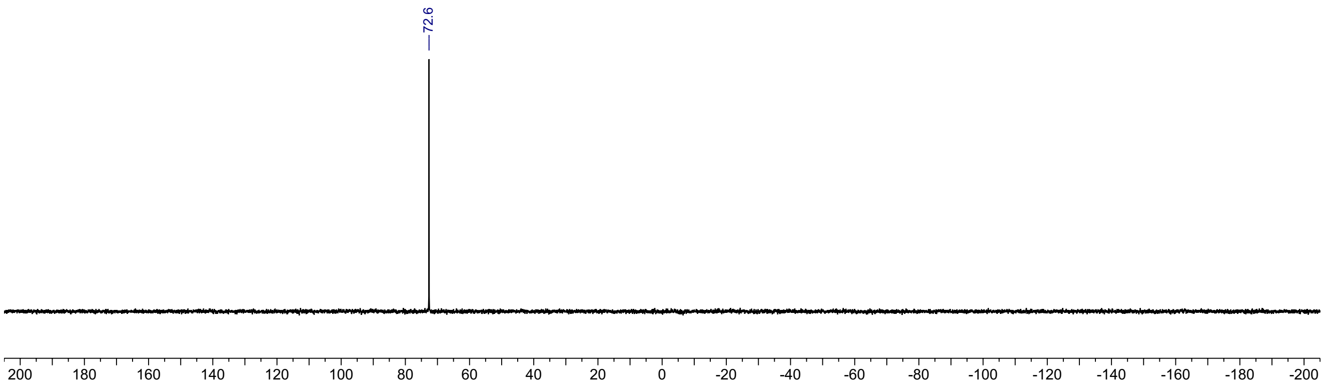

Figure S15. $^{31}P\{^{1}H\}$ NMR spectrum of $[Pd(PAd_3)_2(NO)][BAr^F_4]$ in DFB (162 MHz).

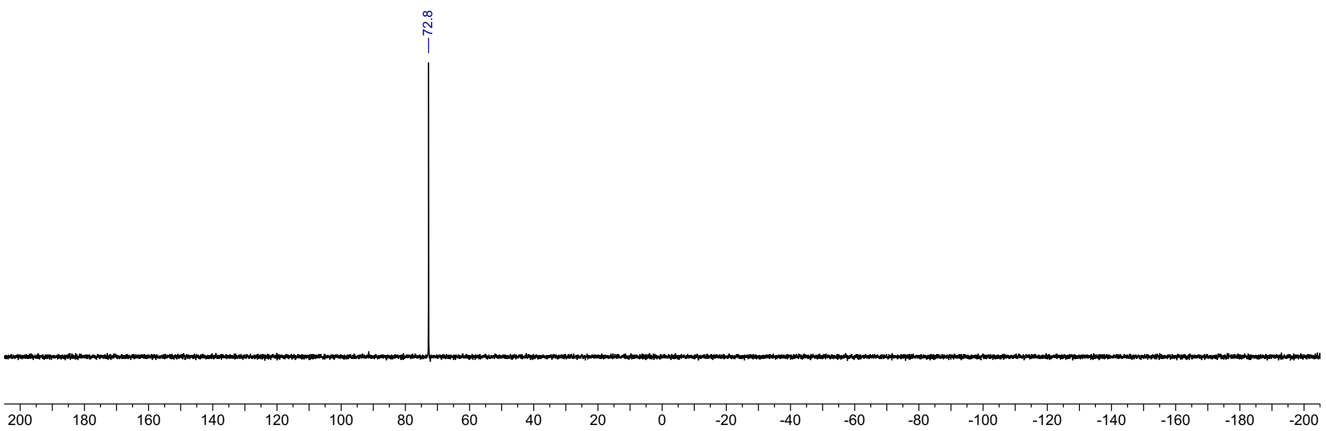

Figure S16. $^{31}P\{^{1}H\}$ NMR spectrum of $[Pd(PAd_3)_2(NO)][BAr^F{}_4]$ in CD_2Cl_2 (162 MHz).

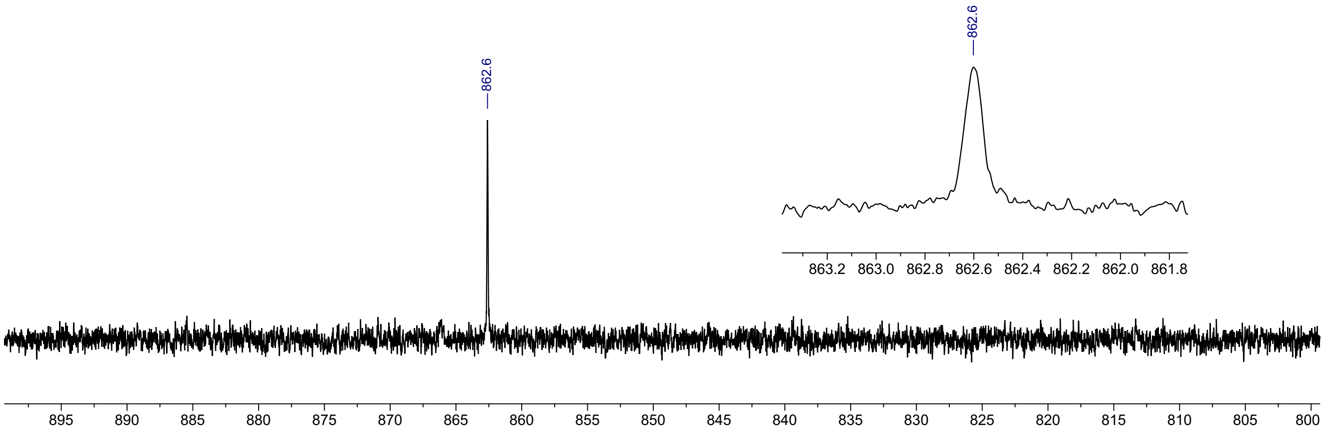

Figure S17. ^{15}N NMR spectrum of $[Pd(PAd_3)_2(NO)][BAr^F{}_4]$ in DFB (61 MHz).

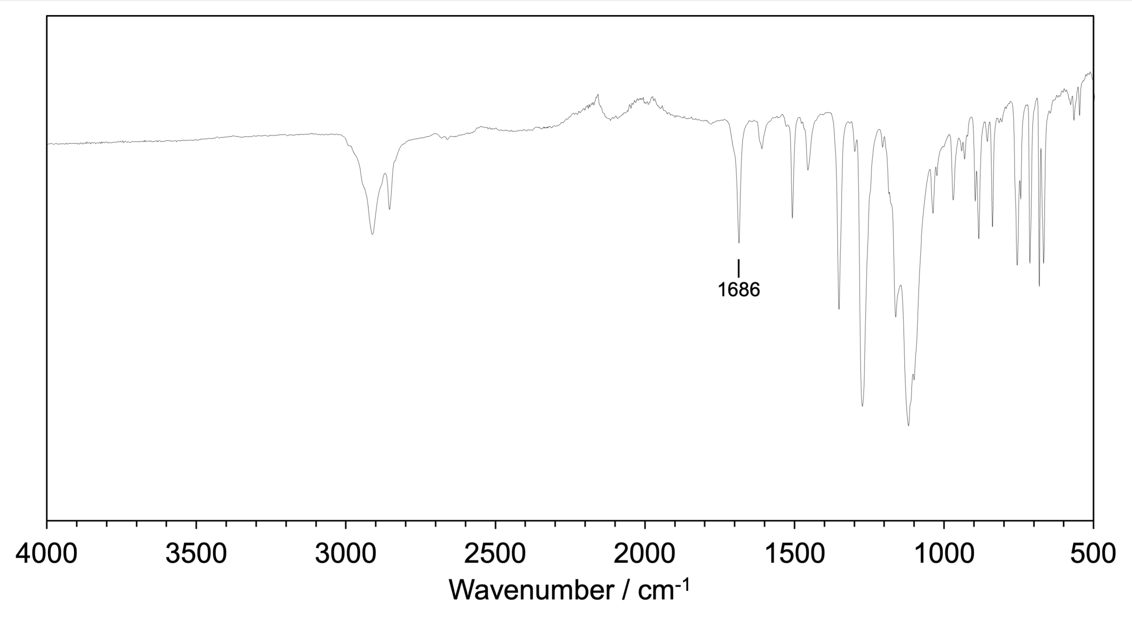

Figure S18. ATR-IR spectrum of $[Pd(PAd_3)_2(NO)][BAr^F{}_4]$.

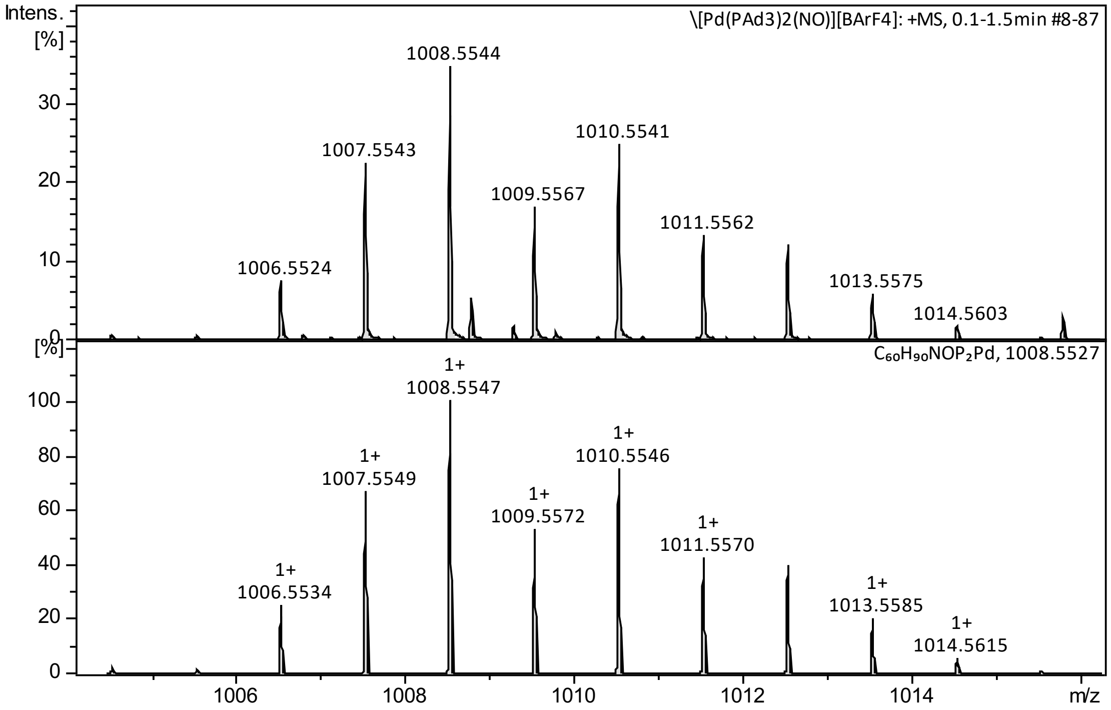

Figure S19. HR ESI-MS of $[Pd(PAd_3)_2(NO)][BAr^F_4]$.

1.7. Preparation and characterisation of $[Pd(PAd_3)_2(NO)][PF_6]$

Following general procedure B using $[Pd(PAd_3)_2]$ (19.6 mg, 20.0 µmol) and $[NO]PF_6$ (3.3 mg, 18.9 µmol, the product was obtained as a dark red crystalline solid. Yield: 11.9 mg (10.3 µmol, 55%).

^{1}H NMR (400 MHz, DFB): δ 2.33 (br, 36H, Ad{2-CH_2}), 1.93 (s, 18H, Ad{3-CH}), 1.71 (d, $^2J_{HH}$ = 12.4, 18H, Ad{4-CH_2}), 1.64 (d, $^2J_{HH}$ = 12.4, 18H, Ad{4-CH_2}).
^{31}P{^{1}H} NMR (162 MHz, DFB): δ 72.6 (s, 2P, PAd_3), -143.2 (hept, $^1J_{PF}$ = 710, 1P, PF_6).
HR ESI-MS (positive ion, 4 kV): 1008.5566 ($[M]^+$, calcd 1008.5547) *m/z*.

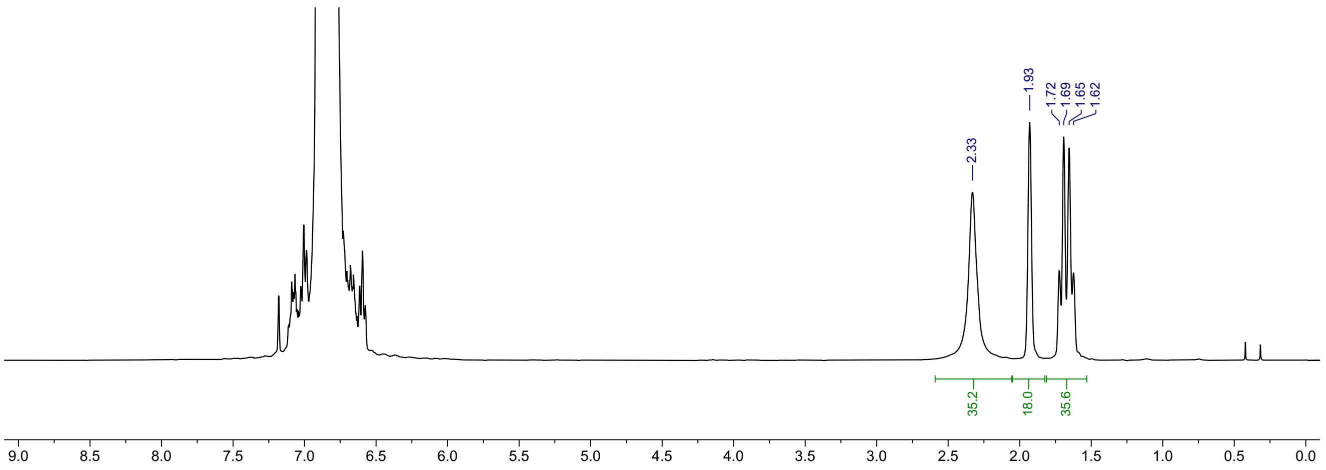

Figure S20. ^{1}H NMR spectrum of $[Pd(PAd_3)_2(NO)][PF_6]$ in DFB (400 MHz).

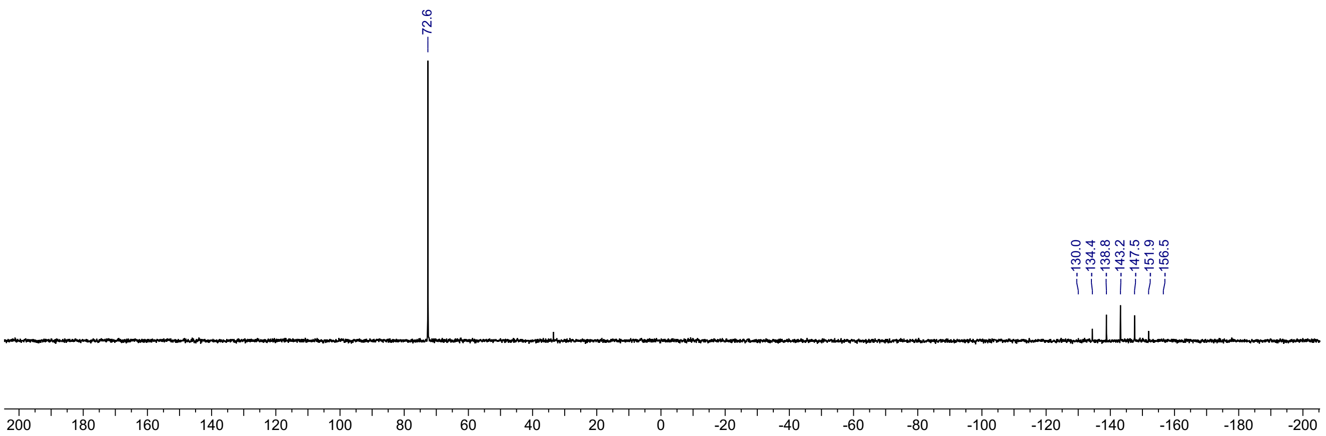

Figure S21. $^{31}P\{^{1}H\}$ NMR spectrum of $[Pd(PAd_3)_2(NO)][PF_6]$ in DFB (162 MHz).

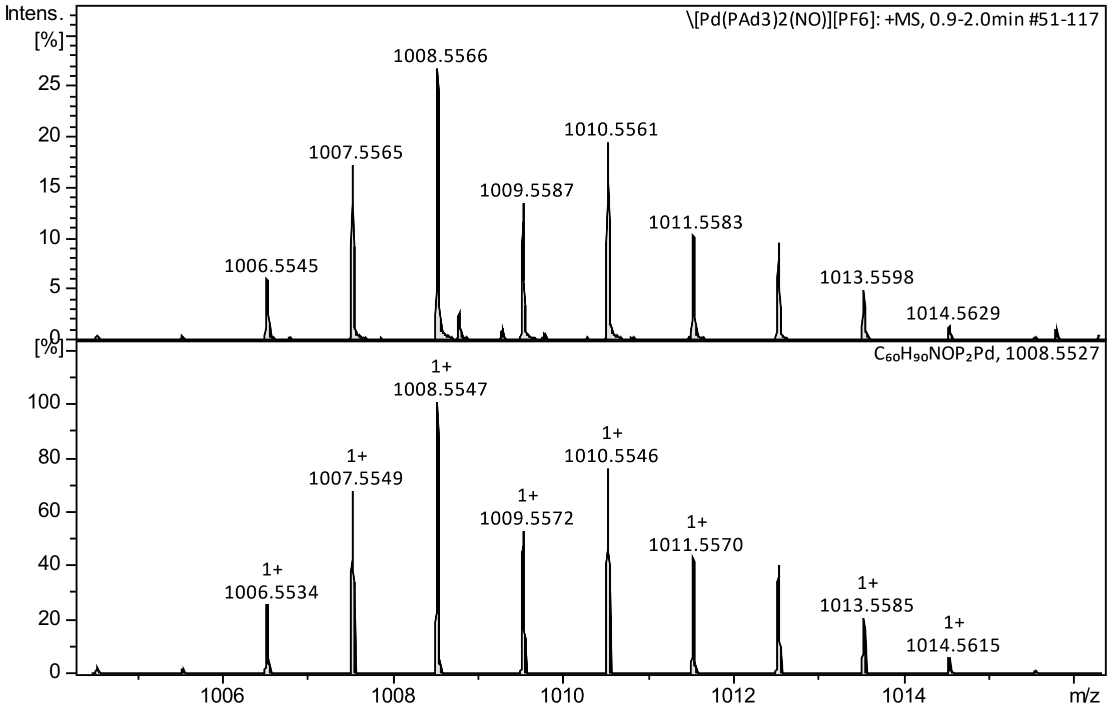

Figure S22. HR ESI-MS of $[Pd(PAd_3)_2(NO)][PF_6]$.

1.8. Preparation and characterisation of [Pt(P*t*Bu$_3$)$_2$(NO)][BAr$^F{}_4$]

Following general procedure A using [Pt(P*t*Bu$_3$)$_2$][BAr$^F{}_4$] (29.3 mg, 20.0 µmol), the product was obtained as a dark green crystalline solid. Yield: 21.5 mg (14.4 µmol, 72%). Following general procedure C using [Pt(P*t*Bu$_3$)$_2$(NO)][PF$_6$] (50.2 mg, 64.8 µmol) and Na[BAr$^F{}_4$] (62.9 mg, 71.0 µmol), the product was obtained as a dark green crystalline solid. Yield: 80.8 mg (54.1 µmol, 84%).

^{1}H NMR (400 MHz, DFB): δ 8.11–8.16 (m, 8H, Ar^F), 7.50 (br, 4H, Ar^F), 1.29 (vt, J_{PH} = 13.5, 54H, *t*Bu).
^{1}H NMR (500 MHz, CD$_2$Cl$_2$): δ 7.71–7.75 (m, 8H, Ar^F), 7.56 (br, 4H, Ar^F), 1.52 (vt, J_{PH} = 13.7, 54H, *t*Bu).
^{13}C{^{1}H} NMR (126 MHz, CD$_2$Cl$_2$): δ 162.3 (q, $^1J_{CB}$ = 50, Ar^F), 135.4 (s, Ar^F), 129.5 (qq, $^2J_{FC}$ = 32, $^2J_{CB}$ = 3, Ar^F), 125.2 (q, $^1J_{FC}$ = 272, Ar^F), 118.0 (sept., $^3J_{FC}$ = 4, Ar^F), 42.2 (vt, J_{PC} = 15, *t*Bu{C}), 32.8 (s, *t*Bu{CH$_3$}).
^{31}P{^{1}H} NMR (162 MHz, DFB): δ 80.3 (s′, $^1J_{PtP}$ = 3983).
^{31}P{^{1}H} NMR (162 MHz, CD$_2$Cl$_2$): δ 80.5 (s′, $^1J_{PtP}$ = 3981).
^{15}N NMR (61 MHz, DFB, ^{15}NO labelled sample): δ 791.3 (t, $^2J_{PN}$ = 3). ^{195}Pt satellites not resolved.
IR (ATR): 1659 (NO) cm^{-1}.
HR ESI-MS (positive ion, 4 kV): 629.3330 ([*M*]$^+$, calcd 629.3326) *m/z*.
Anal. calcd for $C_{56}H_{66}BF_{24}NOP_2Pt$ (1492.95 g·mol^{-1}): C, 45.05; H, 4.46; N, 0.94. Found: C, 45.03; H, 4.31; N, 0.87.

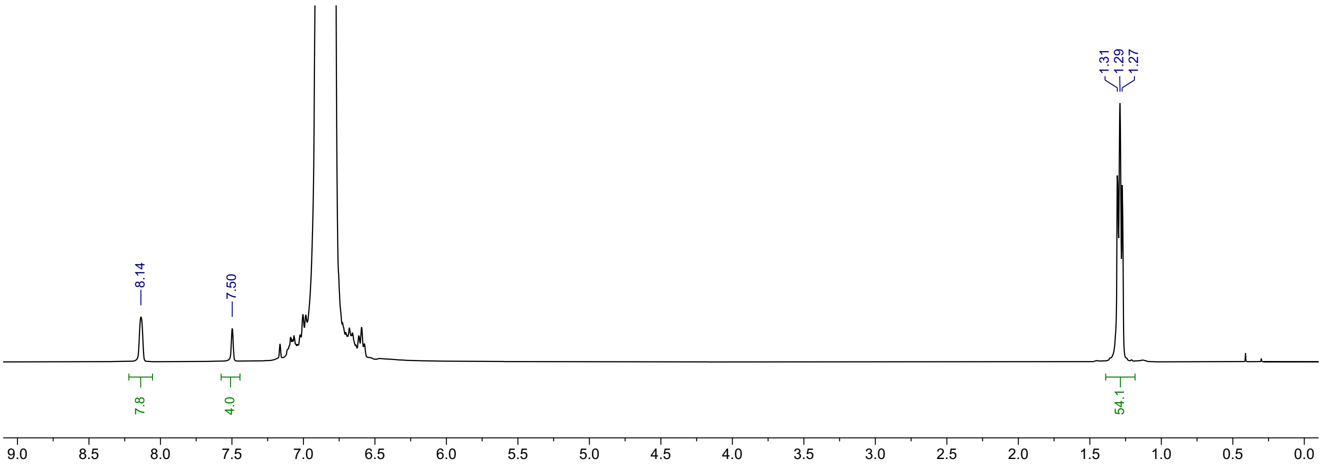

Figure S23. ^{1}H NMR spectrum of [Pt(P*t*Bu$_3$)$_2$(NO)][BAr$^F{}_4$] in DFB (400 MHz).

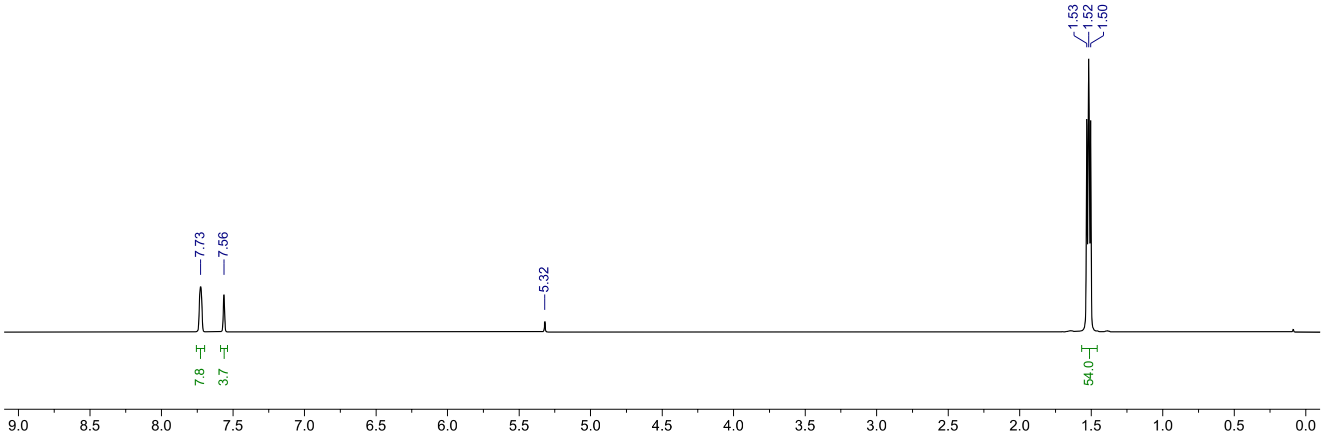

Figure S24. ^1H NMR spectrum of [Pt(P*t*Bu_3)$_2$(NO)][BAr^F_4] in CD_2Cl_2 (500 MHz).

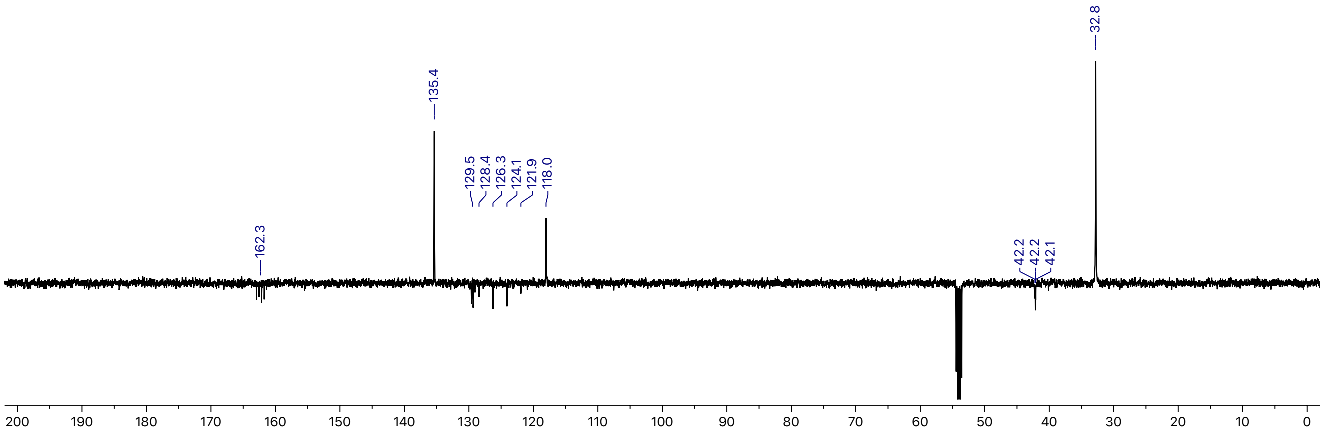

Figure S25. $^{13}C\{^1H\}$ APT NMR spectrum of [Pt(P*t*Bu_3)$_2$(NO)][BAr^F_4] in CD_2Cl_2 (126 MHz).

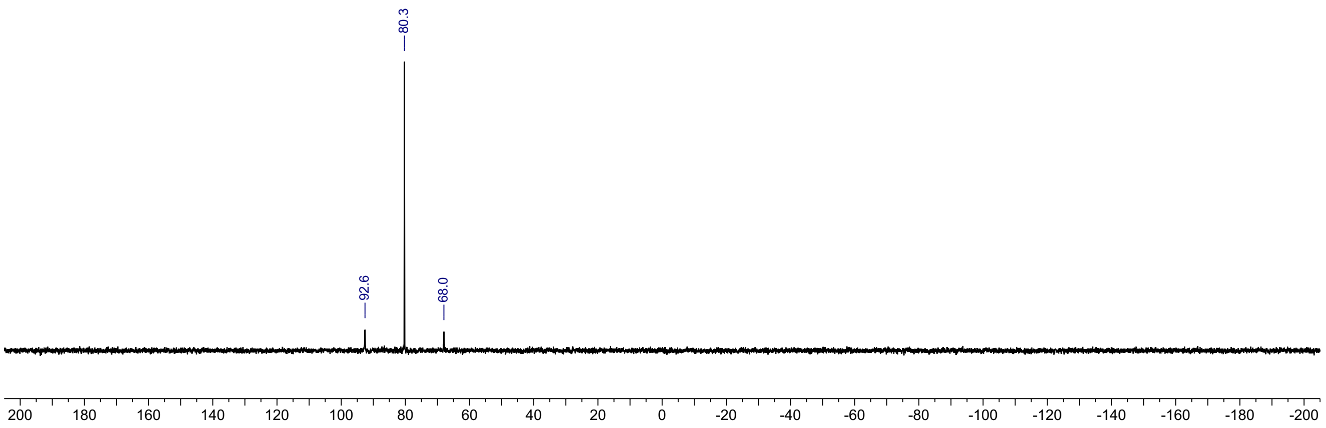

Figure S26. $^{31}P\{^1H\}$ NMR spectrum of [Pt(P*t*Bu_3)$_2$(NO)][BAr^F_4] in DFB (162 MHz).

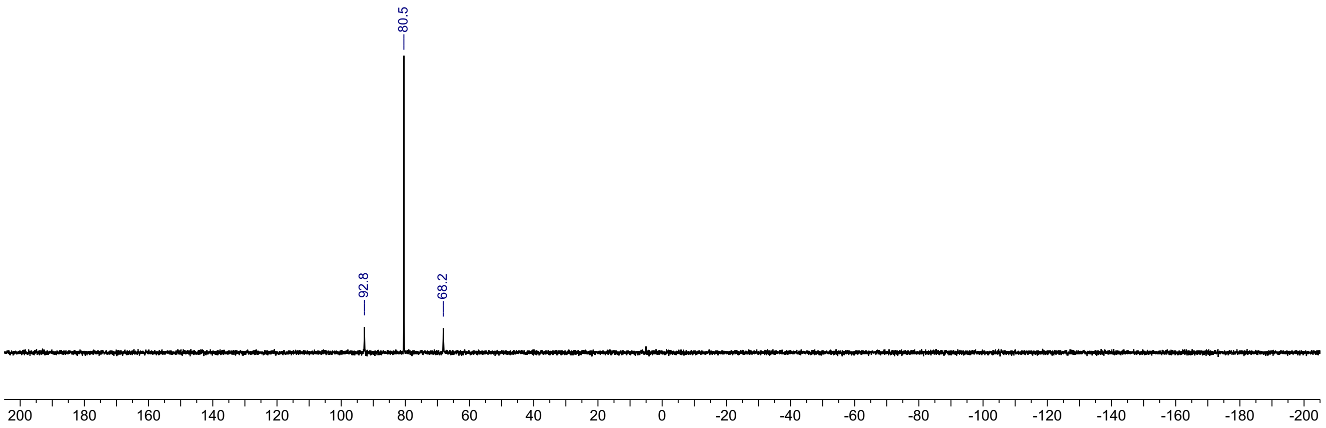

Figure S27. $^{31}P\{^{1}H\}$ NMR spectrum of [Pt(P*t*Bu$_3$)$_2$(NO)][BAr^F$_4$] in CD_2Cl_2 (162 MHz).

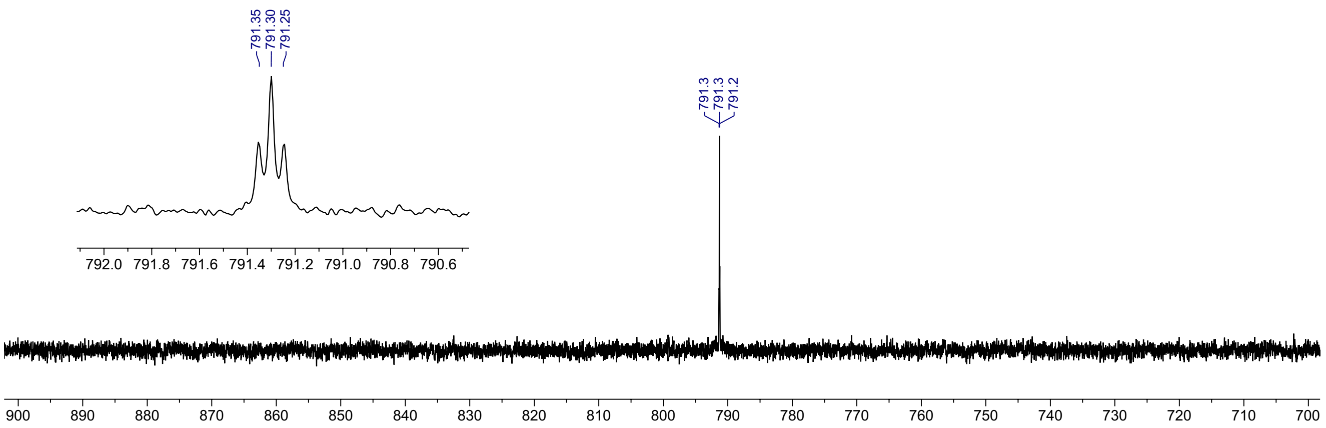

Figure S28. ^{15}N NMR spectrum of [Pt(P*t*Bu$_3$)$_2$(NO)][BAr^F$_4$] in DFB (61 MHz).

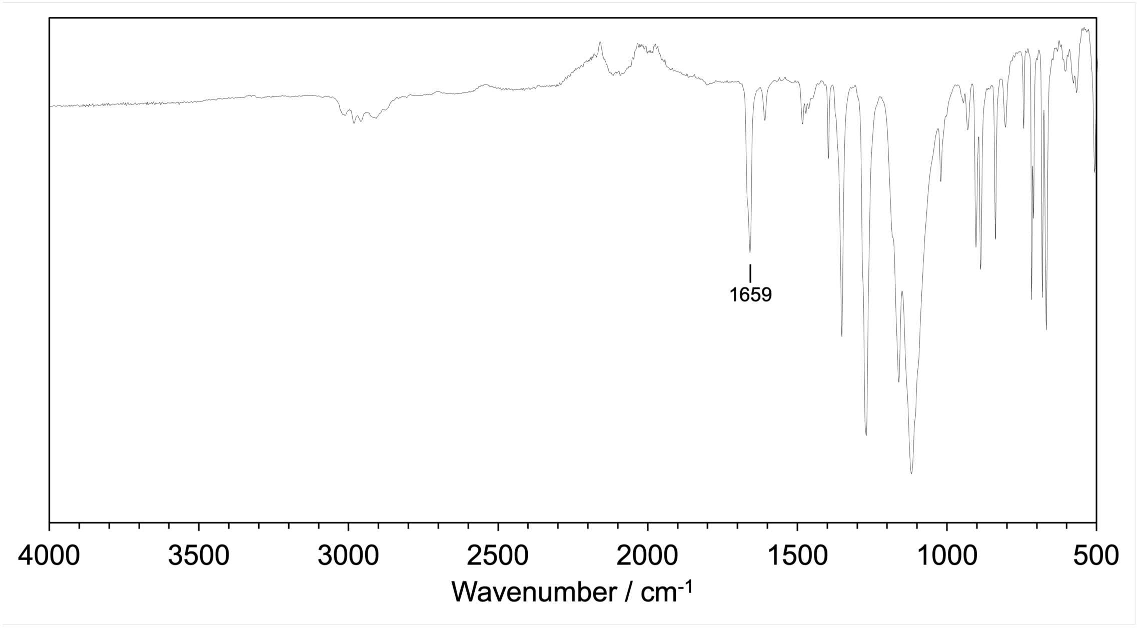

Figure S29. ATR-IR spectrum of [Pt(P*t*Bu$_3$)$_2$(NO)][BAr^F$_4$].

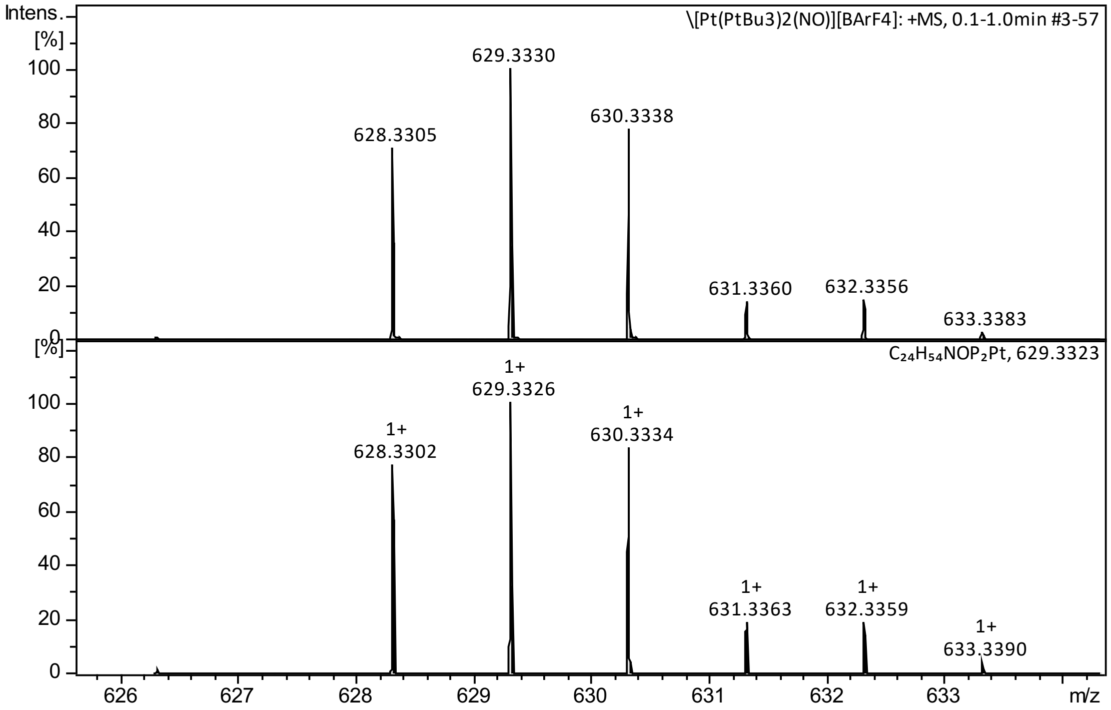

Figure S30. HR ESI-MS of $[Pt(PtBu_3)_2(NO)][BAr^F_4]$.

1.9. Preparation and characterisation of $[Pt(PtBu_3)_2(NO)][PF_6]$

Following general procedure B using $[Pt(PtBu_3)_2]$ (251 mg, 419 μmol) and $[NO]PF_6$ (69.1 mg, 395 μmol), the product was obtained as a dark green crystalline solid. Yield: 273 mg (352 μmol, 89%).

^{1}H NMR (400 MHz, DFB): δ 1.28 (vt, J_{PH} = 13.6, 54H, *t*Bu).
^{31}P{^{1}H} NMR (162 MHz, DFB): δ 80.3 (s′, $^1J_{PtP}$ = 3985, 2P, P*t*Bu3), -143.2 (hept, $^1J_{PF}$ = 710, 1P, PF_6).
HR ESI-MS (positive ion, 4 kV): 629.3327 ([*M*]$^+$, calcd 631.3326) *m/z*.

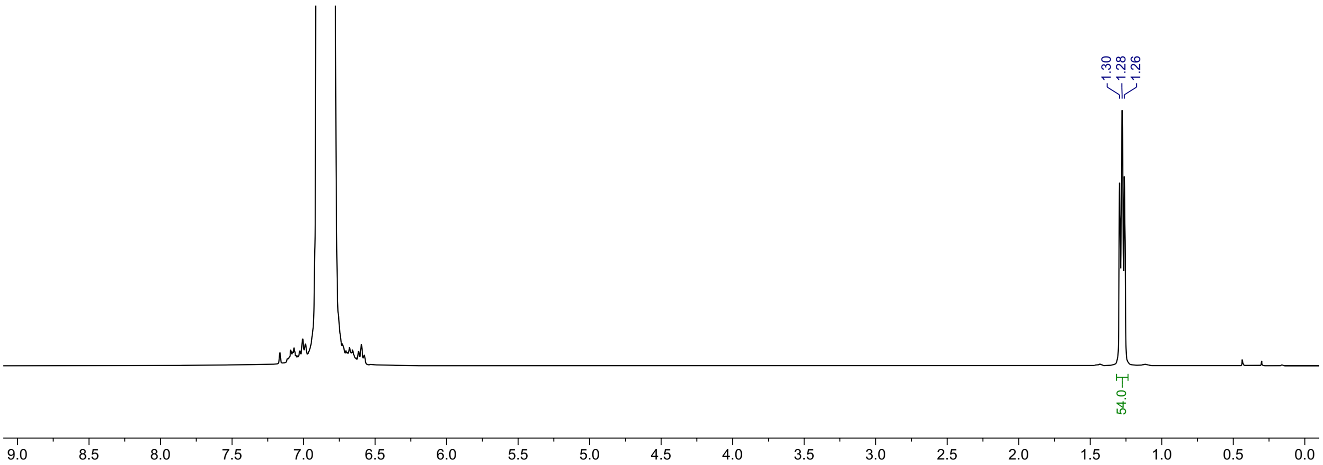

Figure S31. ^{1}H NMR spectrum of $[Pt(PtBu_3)_2(NO)][PF_6]$ in DFB (400 MHz).

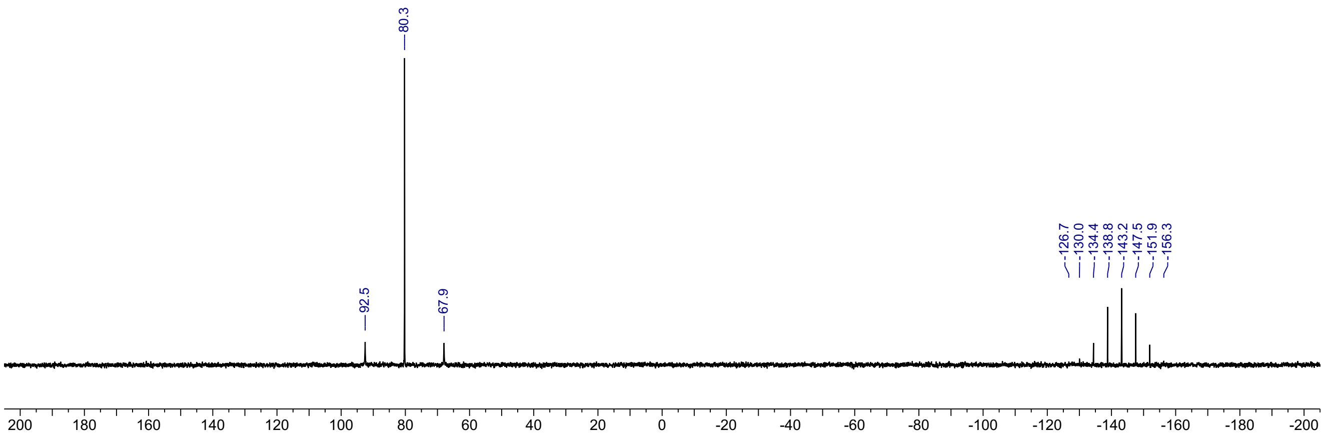

Figure S32. $^{31}P\{^{1}H\}$ NMR spectrum of $[Pt(PtBu_3)_2(NO)][PF_6]$ in DFB (162 MHz).

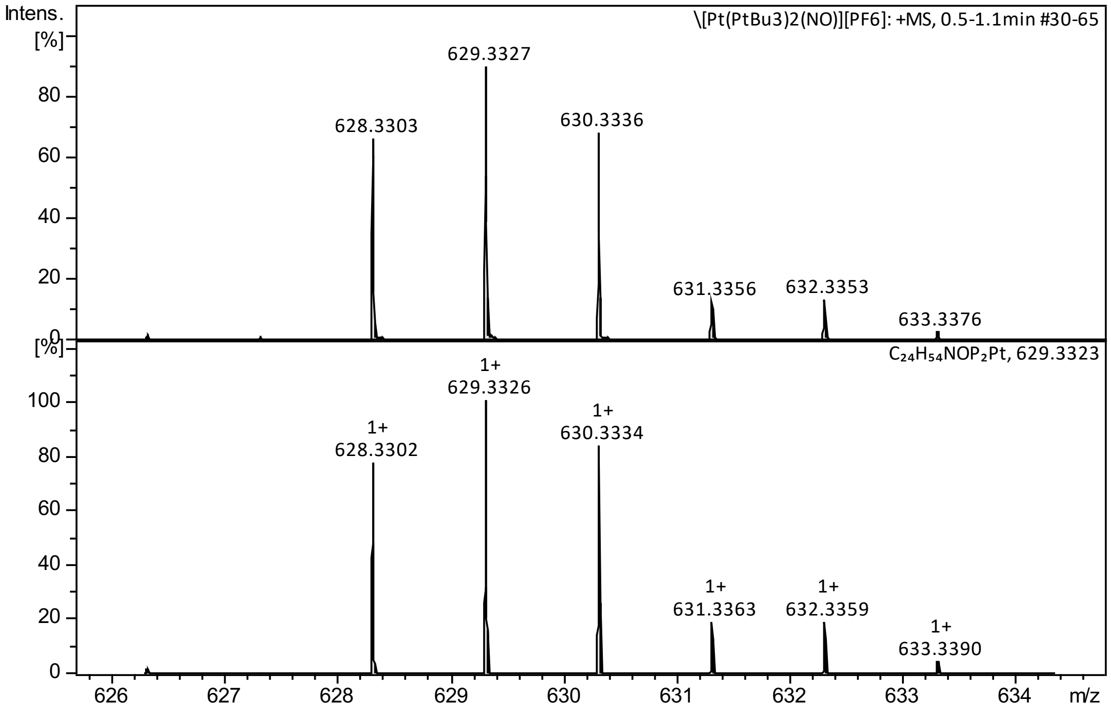

Figure S33. HR ESI-MS of $[Pt(PtBu_3)_2(NO)][PF_6]$.

1.10. Preparation and characterisation of $[Pt(PAd_3)_2(NO)][BAr^F_4]$

Following general procedure A using $[Pt(PAd_3)_2][BAr^F_4]$ (38.7 mg, 20.0 μmol), the product was obtained as a dark green crystalline solid. Yield: 35.1 mg (17.9 μmol, 90%). Following general procedure C using $[Pt(PAd_3)_2(NO)][PF_6]$ (10.2 mg, 8.2 μmol) and $Na[BAr^F_4]$ (8.0 mg, 9.0 μmol), the product was obtained as a dark green crystalline solid. Yield: 11.3 mg (5.8 μmol, 71%).

^{1}H NMR (400 MHz, DFB): δ 8.11–8.17 (m, 8H, Ar^F), 7.50 (br, 4H, Ar^F), 2.44 (br, 36H, Ad{2-CH_2}), 1.95 (s, 18H, Ad{3-CH}), 1.71 (d, $^2J_{HH}$ = 12.5, 18H, Ad{4-CH_2}), 1.64 (d, $^2J_{HH}$ = 12.5, 18H, Ad{4-CH_2}).
^{1}H NMR (600 MHz, CD_2Cl_2): δ 7.71–7.75 (m, 8H, Ar^F), 7.56 (br, 4H, Ar^F), 2.52 (br, 36H, Ad{2-CH_2}), 2.09 (s, 18H, Ad{3-CH}), 1.79 (unresolved AB resonances, 36H, 2×Ad{4-CH_2}).
^{13}C{^{1}H} NMR (151 MHz, CD_2Cl_2): δ 162.3 (q, $^1J_{CB}$ = 50, Ar^F), 135.4 (s, Ar^F), 129.4 (qq, $^2J_{FC}$ = 32, $^2J_{CB}$ = 3, Ar^F), 125.2 (q, $^1J_{FC}$ = 272, Ar^F), 118.0 (sept., $^3J_{FC}$ = 4, Ar^F), 50.8 (vt, J_{PC} = 9, Ad{1-C}), 44.5 (observed in HMBC only, Ad{2-CH_2}), 36.7 (s, Ad{4-CH_2}), 29.9 (vt, J_{PC} = 8, Ad{3-CH}).
^{31}P{^{1}H} NMR (162 MHz, DFB): δ 63.9 (s′, $^1J_{PtP}$ = 3915).
^{31}P{^{1}H} NMR (162 MHz, CD_2Cl_2): δ 64.2 (s′, $^1J_{PtP}$ = 3906).
^{15}N NMR (61 MHz, DFB, ^{15}NO labelled sample): δ 797.0 (t, $^2J_{PN}$ = 2). ^{195}Pt satellites not resolved.
IR (ATR): 1631 (NO) cm^{-1}.
HR ESI-MS (positive ion, 4 kV): 1097.6152 ($[M]^+$, calcd 1097.6145) *m/z*.
Anal. calcd for $C_{92}H_{102}BF_{24}NOP_2Pt$ (1961.64 g·mol^{-1}): C, 56.33; H, 5.24; N, 0.71. Found: C, 56.86; H, 5.00; N, 0.66.

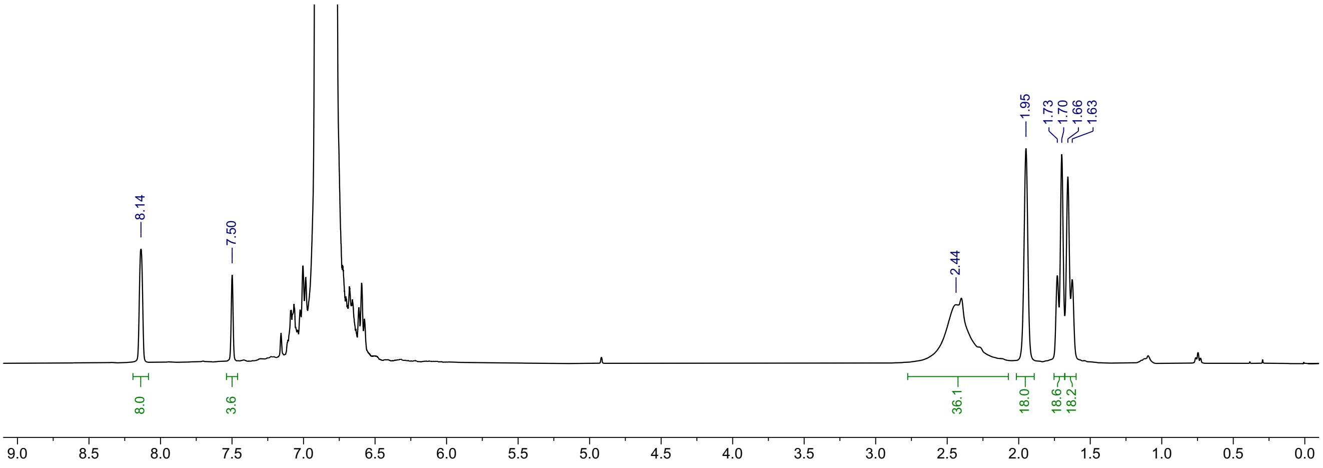

Figure S34. ^{1}H NMR spectrum of $[Pt(PAd_3)_2(NO)][BAr^F_4]$ in DFB (400 MHz).

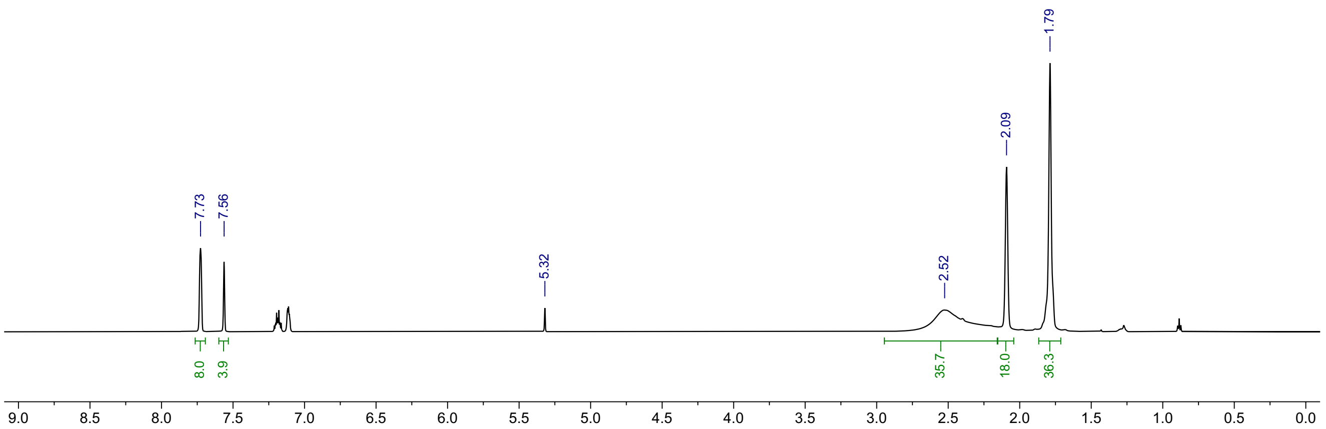

Figure S35. ^{1}H NMR spectrum of $[Pt(PAd_3)_2(NO)][BAr^F{}_4]$ in CD_2Cl_2 (600 MHz). Sample contains residual DFB from recrystallisation.

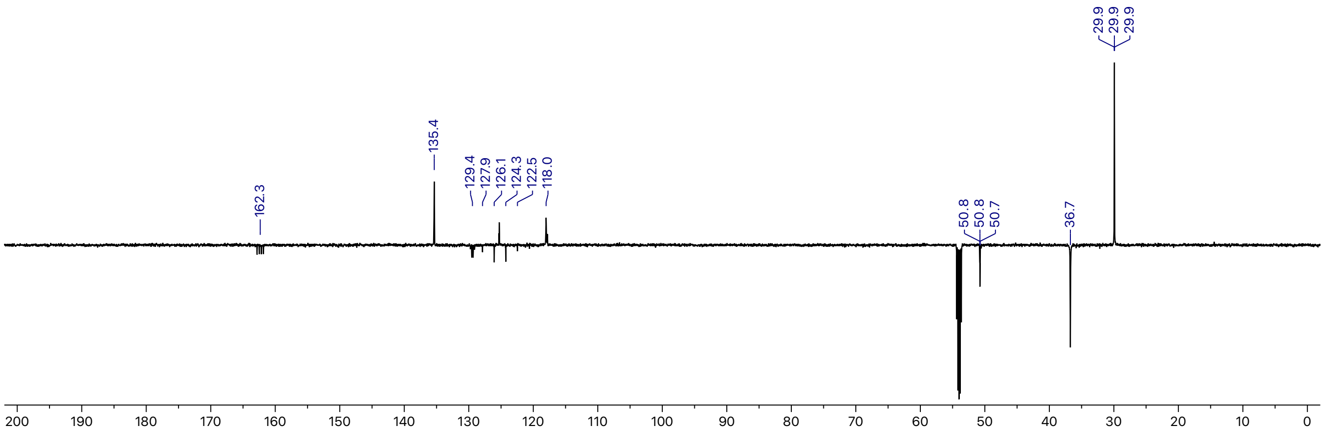

Figure S36. $^{13}C\{^{1}H\}$ APT NMR spectrum of $[Pt(PAd_3)_2(NO)][BAr^F{}_4]$ in CD_2Cl_2 (151 MHz). Sample contains residual DFB from recrystallisation.

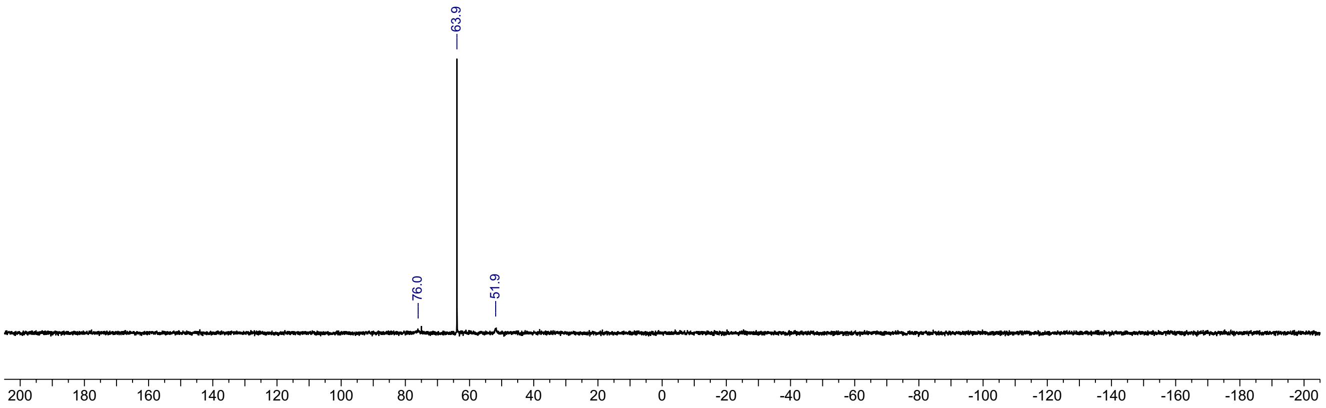

Figure S37. $^{31}P\{^{1}H\}$ NMR spectrum of $[Pt(PAd_3)_2(NO)][BAr^F{}_4]$ in DFB (162 MHz).

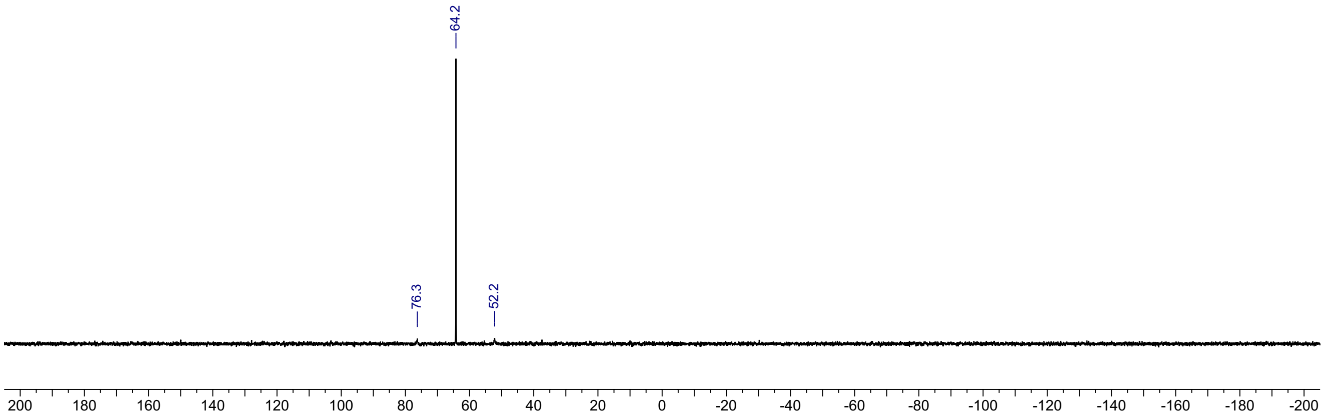

Figure S38. $^{31}P\{^1H\}$ NMR spectrum of $[Pt(PAd_3)_2(NO)][BAr^F_4]$ in CD_2Cl_2 (162 MHz).

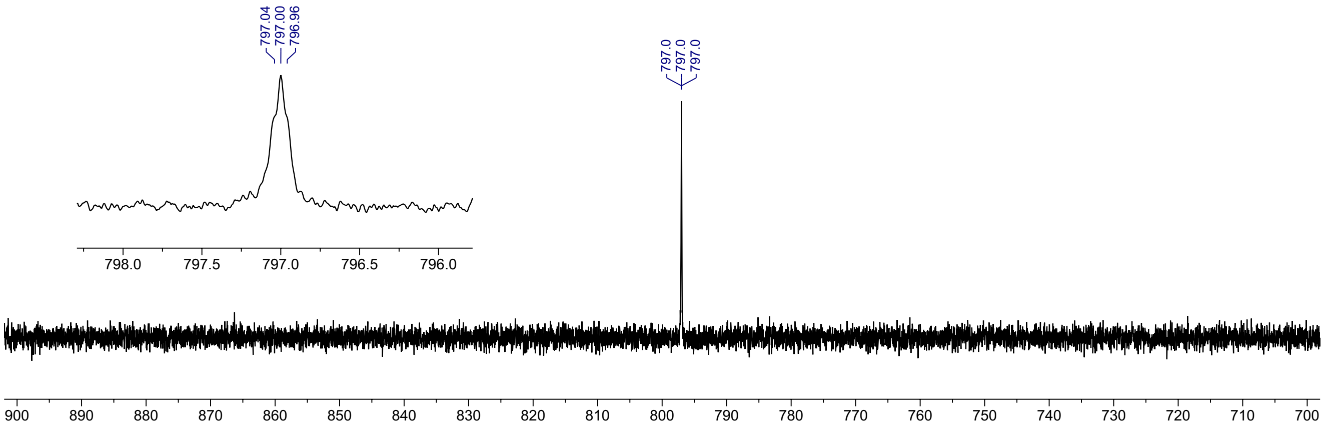

Figure S39. ^{15}N NMR spectrum of $[Pt(PAd_3)_2(NO)][BAr^F_4]$ in DFB (61 MHz).

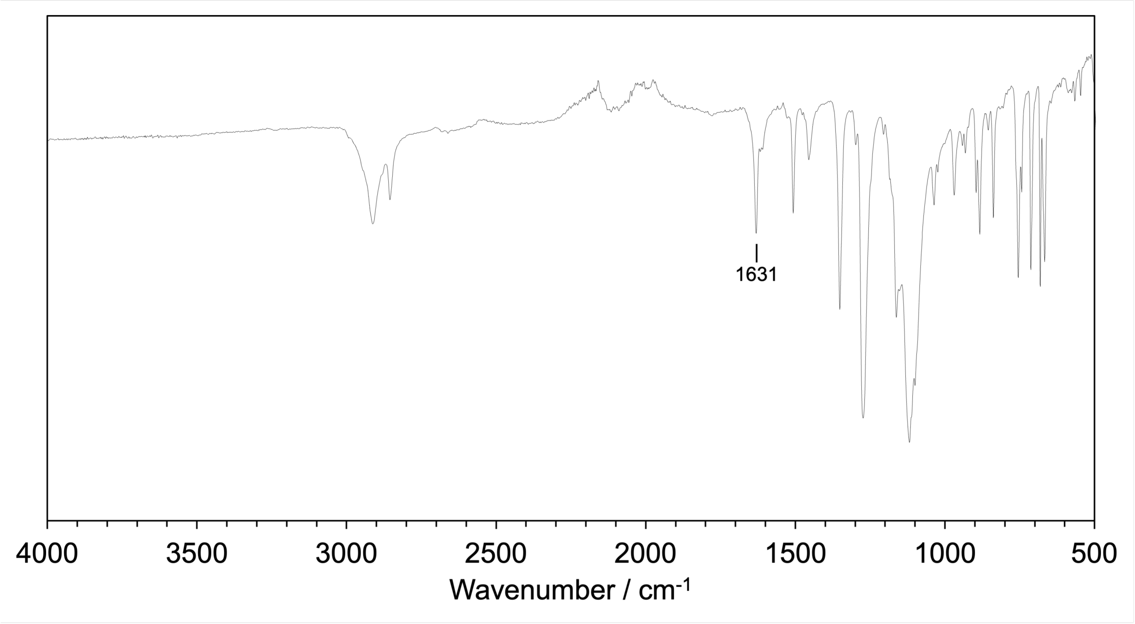

Figure S40. ATR-IR spectrum of $[Pt(PAd_3)_2(NO)][BAr^F_4]$.

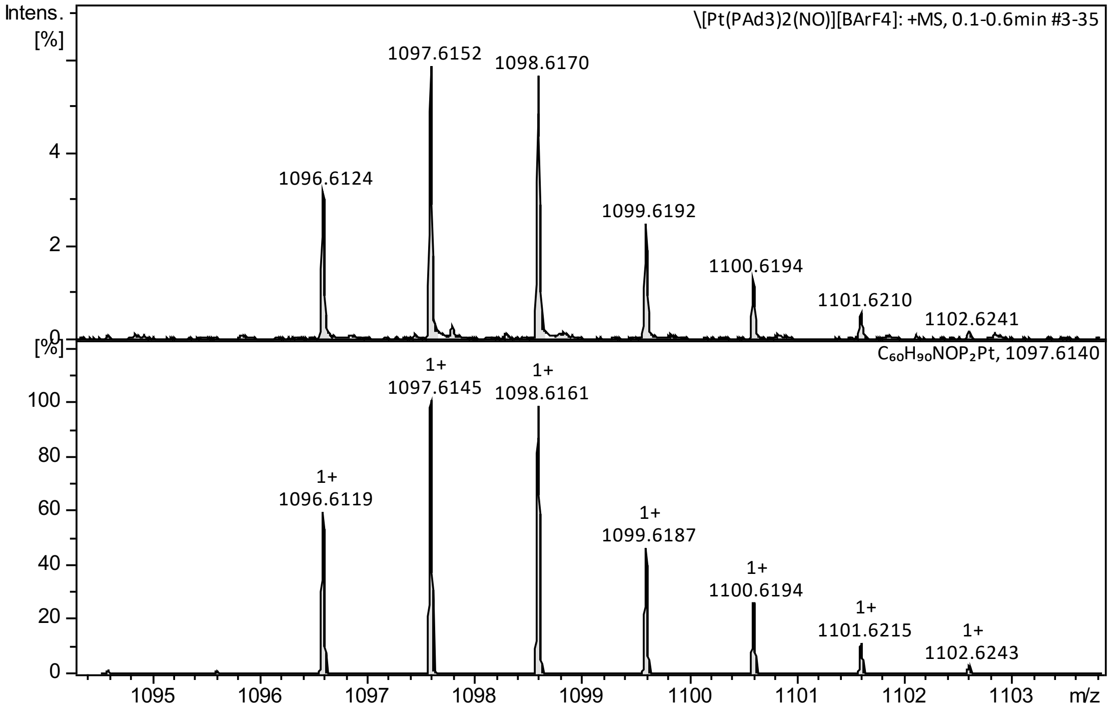

Figure S41. HR ESI-MS of [Pt(PAd$_3$)$_2$(NO)][BAr^F$_4$].

1.11. Preparation and characterisation of [Pt(PAd$_3$)$_2$(NO)][PF$_6$]

Following general procedure B using [Pt(PAd$_3$)$_2$] (21.4 mg, 20.0 µmol) and [NO]PF$_6$ (3.3 mg, 18.9 µmol), the product as obtained as a dark green crystalline solid. Yield: 15.1 mg (12.1 µmol, 64%).

^{1}H NMR (400 MHz, DFB): δ 2.43 (br, 36H, Ad{2-CH$_2$}), 1.95 (s, 18H, Ad{3-CH}), 1.71 (d, $^2J_{HH}$ = 12.3, 18H, Ad{4-CH$_2$}), 1.64 (d, $^2J_{HH}$ = 12.3, 18H, Ad{4-CH$_2$}).
^{31}P{^{1}H} NMR (162 MHz, DFB): δ 63.9 (s′, $^1J_{PtP}$ = 3900, 2P, PAd$_3$), -143.2 (hept, $^1J_{PF}$ = 710, 1P, PF$_6$).
HR ESI-MS (positive ion, 4 kV): 1097.6163 ([*M*]$^+$, calcd 1097.6145) *m/z*.

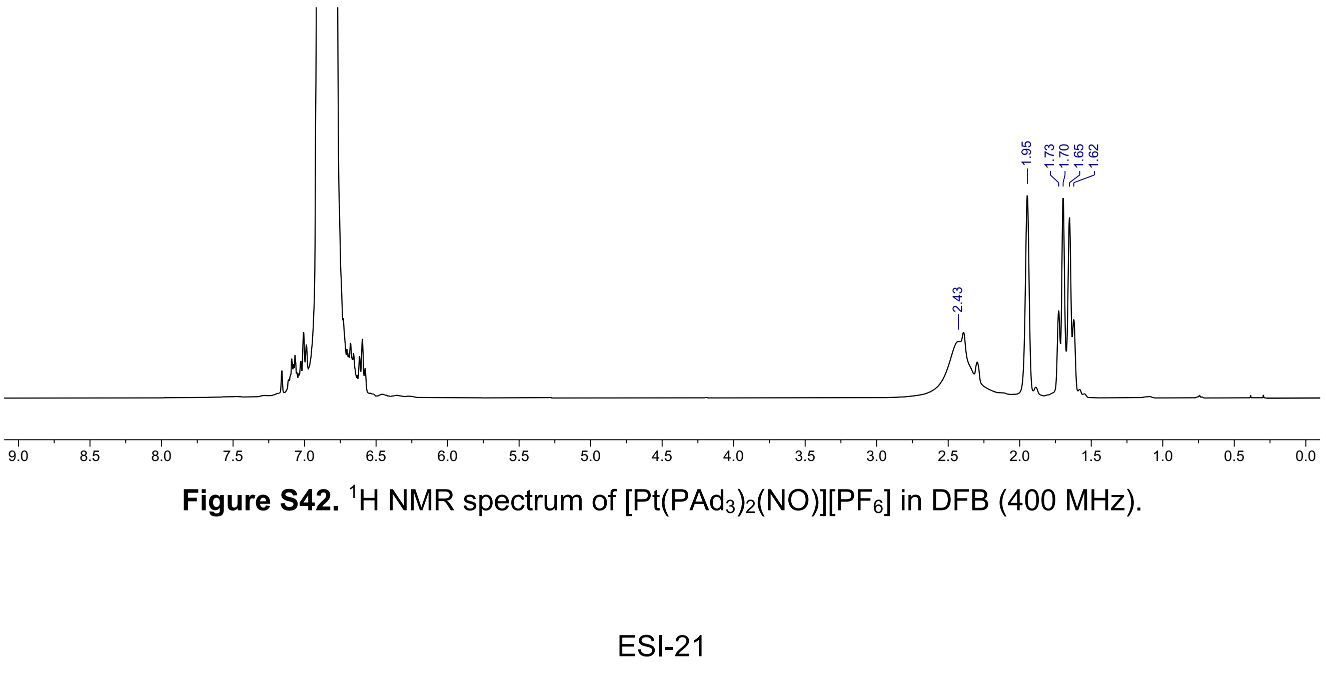

Figure S42. ^{1}H NMR spectrum of [Pt(PAd$_3$)$_2$(NO)][PF$_6$] in DFB (400 MHz).

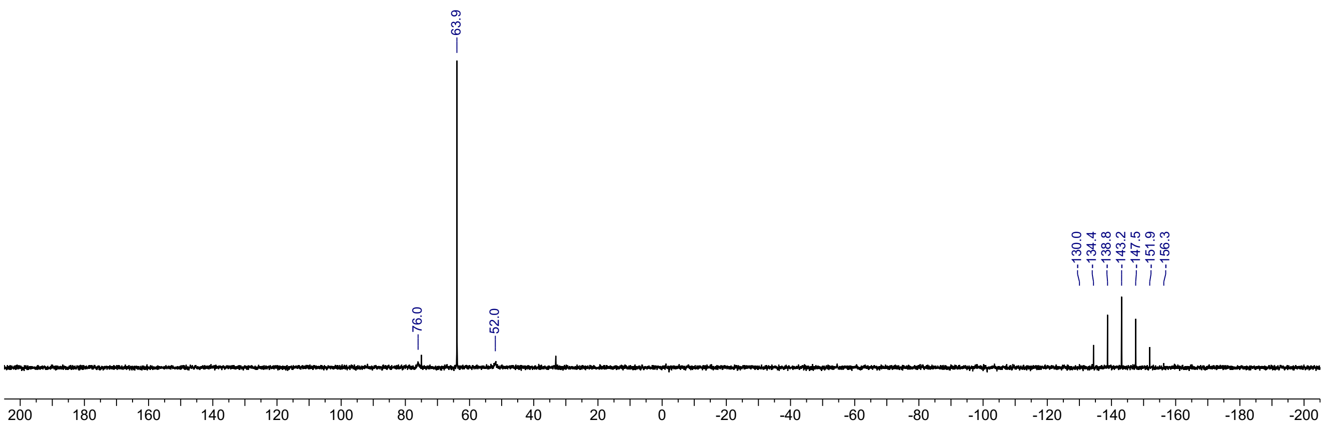

Figure S43. $^{31}P\{^1H\}$ NMR spectrum of $[Pt(PAd_3)_2(NO)][PF_6]$ in DFB (162 MHz).

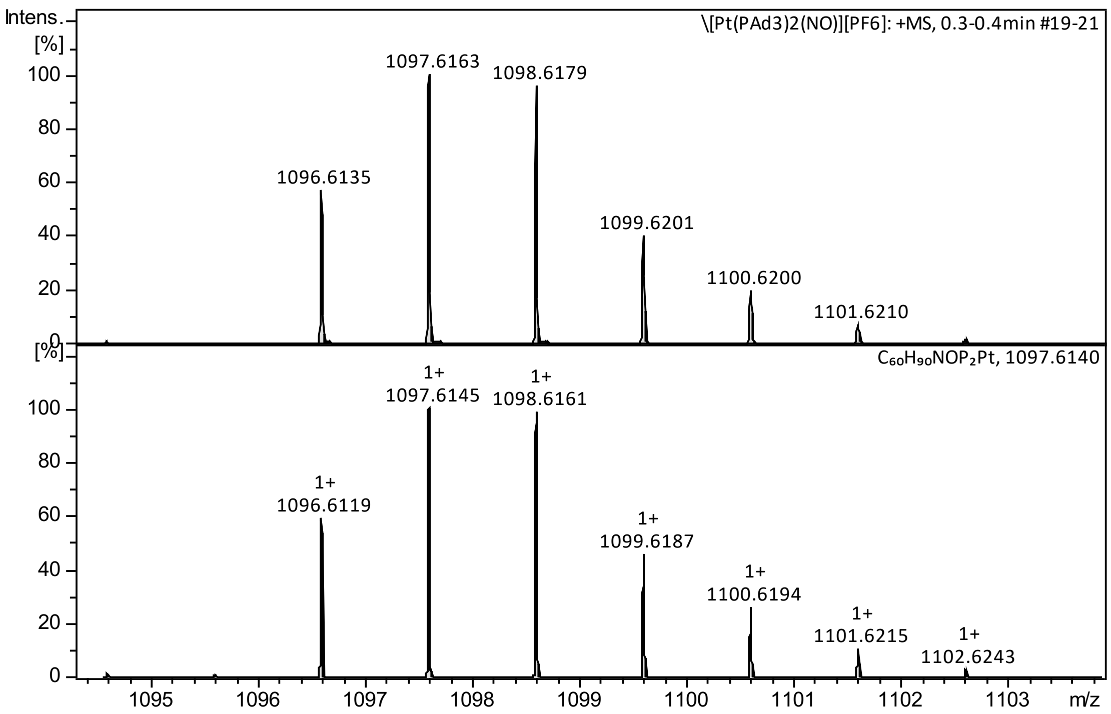

Figure S44. HR ESI-MS of $[Pt(PAd_3)_2(NO)][PF_6]$.

2. Computational details

2.1. General methods

All geometry optimisations and analytical vibrational frequency calculations were carried out using the Gaussian 16 software package (revision A.03).[7] Geometries were optimised starting from the corresponding X-ray structures and employing the M06 functional and the def2-SVP basis set on all atoms.[8,9] This level of theory was selected after benchmarking a range of widely-used functionals (B3LYP-D3BJ, BP86-D3BJ, M06, M06-2X, M06-L) and basis sets (LANL2DZ and def2-SVP). The resulting geometries of complexes **1–4** at the different levels of theory were compared with the X-ray structures, with special attention to the M–N and N–O bond distances as well as the M–N–O angle

(Figure S45). The accuracy of the different functionals and basis sets was assessed by mean of the root mean square deviations (RMSDs). Overall, the Minnesota-type functionals M06 and M06-2X provided geometries in best agreement with the crystallographic data based on all-heavy atom RMSDs. Regarding the basis set, the relativistic Ahlrichs def2-SVP outperforms the LANL2DZ/6-31G** basis set. All optimised stationary points were characterised by harmonic frequency calculations at the same level of theory, with minima having only positive vibrational frequencies. Additional single point energy calculations were carried out at the M06/def2-TZVP level of theory, from which the wavefunctions were extracted for further analysis (*vide infra*). Coordinates of the complexes at the M06/def2-SVP level of theory are provided in XYZ format.

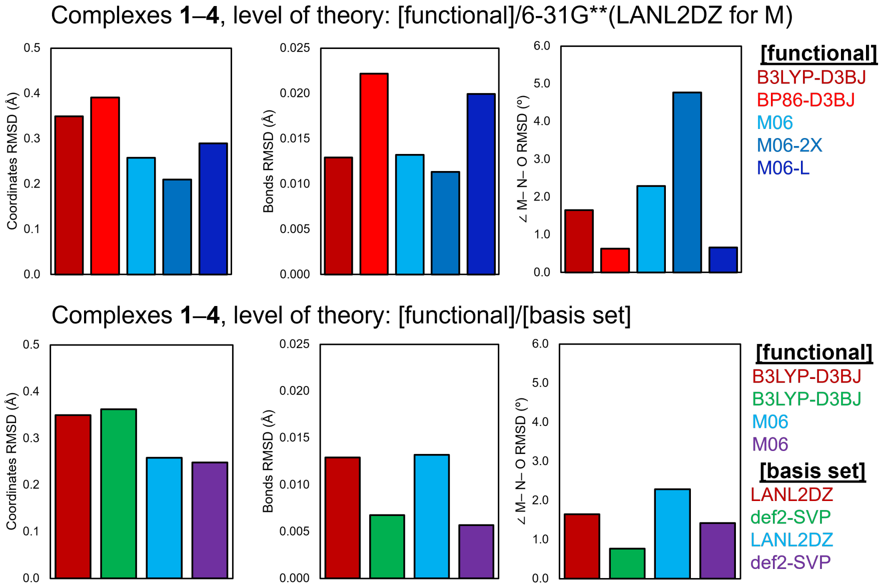

Figure S45. RMSD results from comparing optimised **1–4** with their respective X-ray structures: (top) accuracy of the different functionals studied with the LANL2DZ basis set for palladium and platinum and the 6-31G** basis set for the rest of the atom; (bottom) accuracy of the B3LYP-D3BJ and M06 functionals with different basis sets.

2.2. Effective oxidation state calculations

Complexes **1–4** were analysed using the Effective Oxidation State (EOS) method[10] as implemented in the APOST-3D software program (Version 4).[11] The topological fuzzy Voronoi cells (TFVC) atomic definition[12] and M06/def2-TZVP//M06/def2-SVP wavefunctions were used for all complexes (Table S1). Results obtained using this level of theory were corroborated by comparison to calculations at the BP86-D3BJ/def2-TZVP//BP86-D3BJ/def2-SVP level of theory,[13] as employed in a recent computational analysis of the coordination polymer $[PdCl(NO)]_n$ (Table S2).[14]

Effective Fragment Orbitals (EFOs) were calculated for the metal and the three ligands (NO and the phosphines). In all cases, the EFOs corresponding to two π^* orbitals of the NO ligand and the five *d*–orbitals of the metal could be identified. Out of these, the d_{Z^2} and d_{XZ} metal EFOs enable bonding interactions with the $\pi^*_{(\perp)}$ and $\pi^*_{(\parallel)}$ NO EFOs, respectively (Figure S46). The symmetry and population of the EFOs helps to characterise the nature of these bonds. The $d_{Z^2}/\pi^*_{(\perp)}$ interaction can be described as a highly polarised σ-bond, whilst the d_{XZ}–$\pi^*_{(\parallel)}$ interaction corresponds to metal-to-ligand π-backdonation and is comparatively more metal-centred. From the occupancies of the EFOs, the percentage of NO character in the bonding interactions can be estimated using the following equation:[15]

$$\%\mathrm{NO} = 100 \cdot \frac{\lambda_{\pi-\mathrm{NO}}}{\lambda_{\pi-\mathrm{NO}} + \lambda_{d-\mathrm{M}}} \qquad \textbf{Eq. S1}$$

where $\lambda_{\pi-\mathrm{NO}}$ and $\lambda_{d-\mathrm{M}}$ are the occupations of the respective EFOs. The reliability index *R*(%) is used to gauge how well the EFOs model the electronic structure of the molecule of interest and is defined:

$$R(\%) = 100 \cdot \min\left(1,\ \max\left(0,\ \lambda^{\varphi}_{\mathrm{HO}} - \lambda^{\varphi}_{\mathrm{LU}} + 0.5\right)\right) \qquad \textbf{Eq. S2}$$

where λ_{HO} and λ_{LU} are the occupancies of the formally highest occupied and lowest unoccupied EFO of a particular spin φ and *R* ranges from 0 to 100. Large *R* indices indicate that the EFO describe the electronic distribution of the wavefunction well. For more detailed information about this technique, the reader is directed to ref. 10.

Table S1. EOS analysis of **1–4** at the M06/def2-TZVP//M06/def2-SVP level of theory.

	**OS**		***R*(%)**	**EFO population**					
	**M**	**NO**		d_{Z^2}	$\pi^*_{(\perp)}$	**%NO**	d_{XZ}	$\pi^*_{(\parallel)}$	**%NO**
**1**	0	+1	76.9	0.632	0.363	36.5	0.787	0.162	17.1
**2**	0	+1	76.6	0.631	0.365	36.6	0.785	0.170	17.8
**3**	0	+1	68.1	0.572	0.391	40.6	0.750	0.172	18.7
**4**	0	+1	67.3	0.569	0.396	41.0	0.747	0.179	19.3

Table S2. EOS analysis of **1–4** at the BP86-D3BJ/def2-TZVP//BP86-D3BJ/def2-SVP level of theory.

	**OS**		***R*(%)**	**EFO population**					
	**M**	**NO**		d_{Z^2}	$\pi^*_{(\perp)}$	**%NO**	d_{XZ}	$\pi^*_{(\parallel)}$	**%NO**
**1**	0	+1	79.6	0.644	0.347	35.0	0.754	0.240	24.1
**2**	0	+1	78.8	0.640	0.352	35.5	0.752	0.229	23.3
**3**	0	+1	72.3	0.593	0.370	38.4	0.722	0.220	23.4
**4**	0	+1	71.2	0.589	0.376	39.0	0.721	0.227	23.9

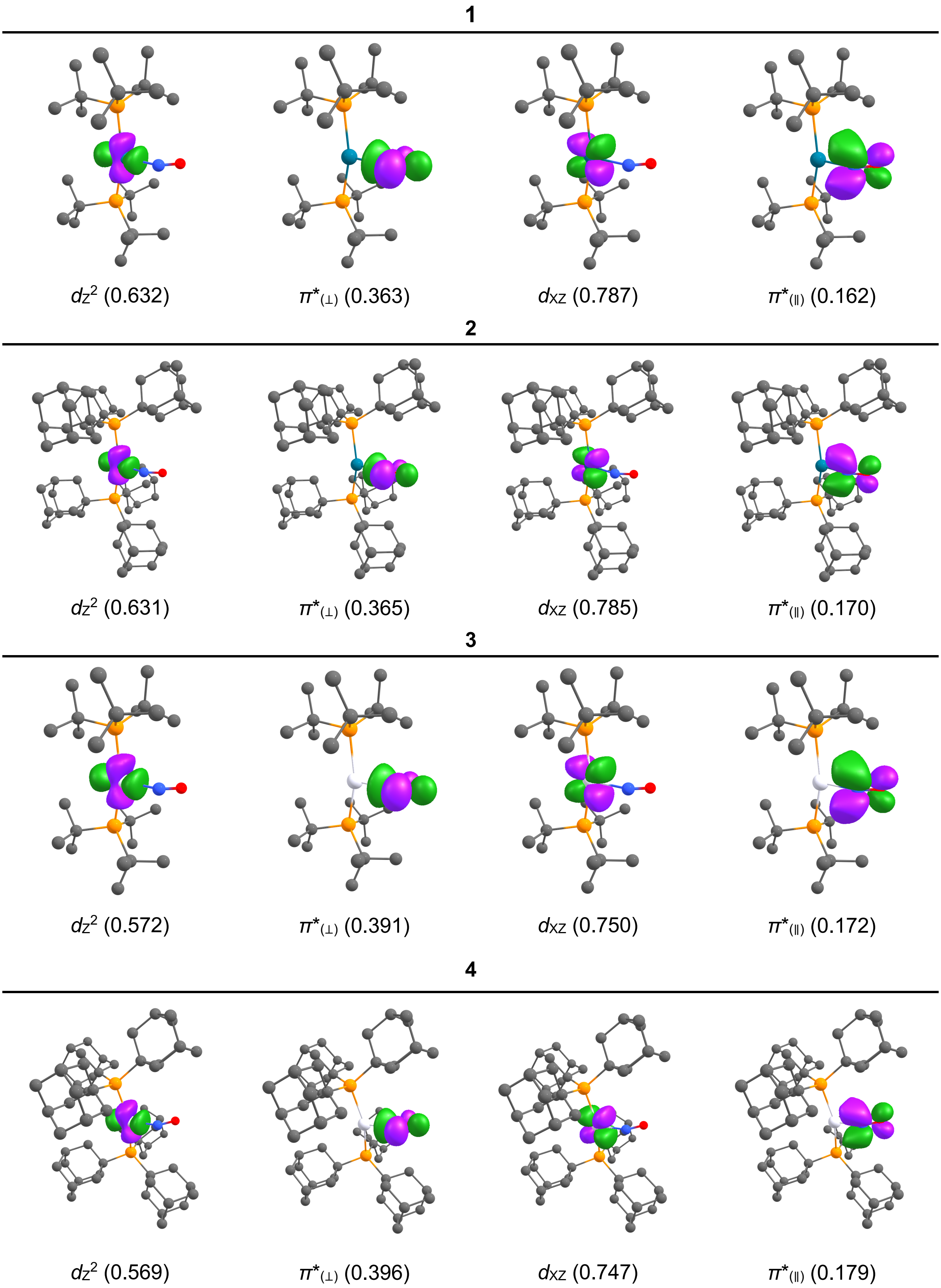

Figure S46. EFOs relevant to the M–NO bonds in **1–4**, with occupancies in parentheses.

2.3. Energy decomposition analysis

To gain a deeper understanding into the nature of the M–NO bond in **1–4**, an energy decomposition analysis (EDA)[16] was conducted using the extended transition state (ETS) method in combination with natural orbitals for chemical valence (NOCV) theory.[17] In this scheme, the interaction energy (ΔE_{int}) between two chemical fragments is deconvoluted into three chemically meaningful contributions: the electrostatic interaction (ΔV_{elstat}), the Pauli repulsion (ΔE_{Pauli}), and the orbital interaction (ΔE_{orb}):

$$\Delta E_{\mathrm{int}} = \Delta V_{\mathrm{elstat}} + \Delta E_{\mathrm{Pauli}} + \Delta E_{\mathrm{orb}} \qquad \textbf{Eq. S3}$$

The electrostatic interaction ΔV_{elstat} corresponds to classical electrostatic interaction between the unperturbed charge distributions of the deformed reactants and is usually attractive. The Pauli repulsion ΔE_{Pauli} comprises the destabilizing interaction between occupied closed-shell orbitals of both deformed reactants due to the Pauli principle. The orbital interaction ΔE_{oi} accounts for polarization and charge transfer between the fragments.

The orbital interaction term can be further decomposed into pairwise contributions of interacting orbitals of the two fragments using the NOCV analysis.[17b] In this approach, the deformation density $\Delta\rho(\mathrm{r})$ is expressed in terms of complementary eigenfunctions (ψ_k ψ_{-k}) with the eigenvalues ν_k and ν_{-k} with the same absolute value but opposite sign:

$$\Delta\rho(r) = \sum_k \upsilon_k\left[-\psi_{-k}^2(r) + \psi_k^2(r)\right] = \sum_k \Delta\rho_k(r) \qquad \textbf{Eq. S4}$$

To obtain the dissociation energy (D_e), it is important to take into account the relaxation of the fragments from their equilibrium structure. This results into the preparation energy (ΔE_{prep}) that can be further decomposed into the preparation energy for each fragment.

$$\Delta E_{\mathrm{prep}} = E_{\mathrm{frag\_A}}^{\mathrm{complex}} - E_{\mathrm{frag\_A}}^{\mathrm{optimised}} + E_{\mathrm{frag\_B}}^{\mathrm{complex}} - E_{\mathrm{frag\_B}}^{\mathrm{optimised}} \qquad \textbf{Eq. S5}$$

By definition, the dissociation energy corresponds to the negative value of the total bonding energy:

$$-D_e = \Delta E_{\mathrm{int}} - \Delta E_{\mathrm{prep}} \qquad \textbf{Eq. S6}$$

ETS-NOCV calculations were carried out using the Amsterdam Density Functional (ADF), AMS2020.102 software package[18] using the Gaussian16 optimised geometries. To align with the level of theory used for the optimisation, the functional M06 was used in conjunction with a triple-ζ quality basis set with two sets of polarisation functions per atom, namely TZ2P.[19] Scalar relativistic effects were included using the zeroth-order regular approximation (ZORA).[20] Numerical quality was set to very good in all calculations.

Informed by the EOS analysis, $\{M(PR_3)_2\}/NO^+$ fragmentation of the metal–nitrosyl bond was selected to carry out the EDA of **1–4**. To check the effect of the M–N interfragment distance on the different energy components, EDA was also performed for variants **1′–4′** where this distance was constrained to the average M–N distance from the optimised geometries of **1–4** (1.915 Å). The data of both EDA analysis are very similar.

Table S3. EDA-NOCV analysis for $\{M(PR_3)_2\}/NO^+$ fragmentation of **1–4** and **1′–4′**. (energies in kcal·mol^{-1}).

	**1**	**2**	**3**	**4**
ΔE_{int}	-130.7	-139.6	-139.8	-149.4
ΔE_{Pauli}	+206.7	+210.3	+253.4	+265.3
ΔV_{elstat}	-114.2	-117.4	-135.5	-142.1
ΔE_{orb}	-223.2	-232.5	-257.7	-272.6
ΔE_{σ} **(%)**	-156.6 (64.4)	-158.4 (62.7)	-177.1 (65.1)	-182.1 (63.5)
ΔE_{π} **(%)**	-50.2 (20.6)	-53.3 (21.1)	-51.6 (19.0)	-56.2 (19.6)
ΔE_{rest} **(%)**	-36.6 (15.0)	-40.7 (16.2)	-43.4 (15.9)	-48.3 (16.9)
ΔE_{prep}	+22.5	+22.3	+26.3	+26.9
ΔE_{bind} **(=** $-D_e$**)**	-108.2	-117.3	-113.5	-122.5

	**1′**	**2′**	**3′**	**4′**
ΔE_{int}	-130.6	-139.5	-140.5	-149.5
ΔE_{Pauli}	+204.8	+204.1	+267.7	+268.2
ΔV_{elstat}	-113.3	-114.9	-141.5	-143.3
ΔE_{orb}	-222.0	-228.8	-266.6	-274.4
ΔE_{σ} **(%)**	-156.0 (64.4)	-156.6 (63.0)	-181.5 (64.6)	-183.0 (63.5)
ΔE_{π} **(%)**	-49.7 (20.5)	-51.9 (20.9)	-54.7 (19.5)	-56.7 (19.7)
ΔE_{rest} **(%)**	-36.4 (15.0)	-40.2 (16.2)	-44.9 (16.0)	-48.7 (16.9)
ΔE_{prep}	+22.4	+22.2	+26.9	+27.0
ΔE_{bind} **(=** $-D_e$**)**	-108.2	-117.2	-113.5	-122.5

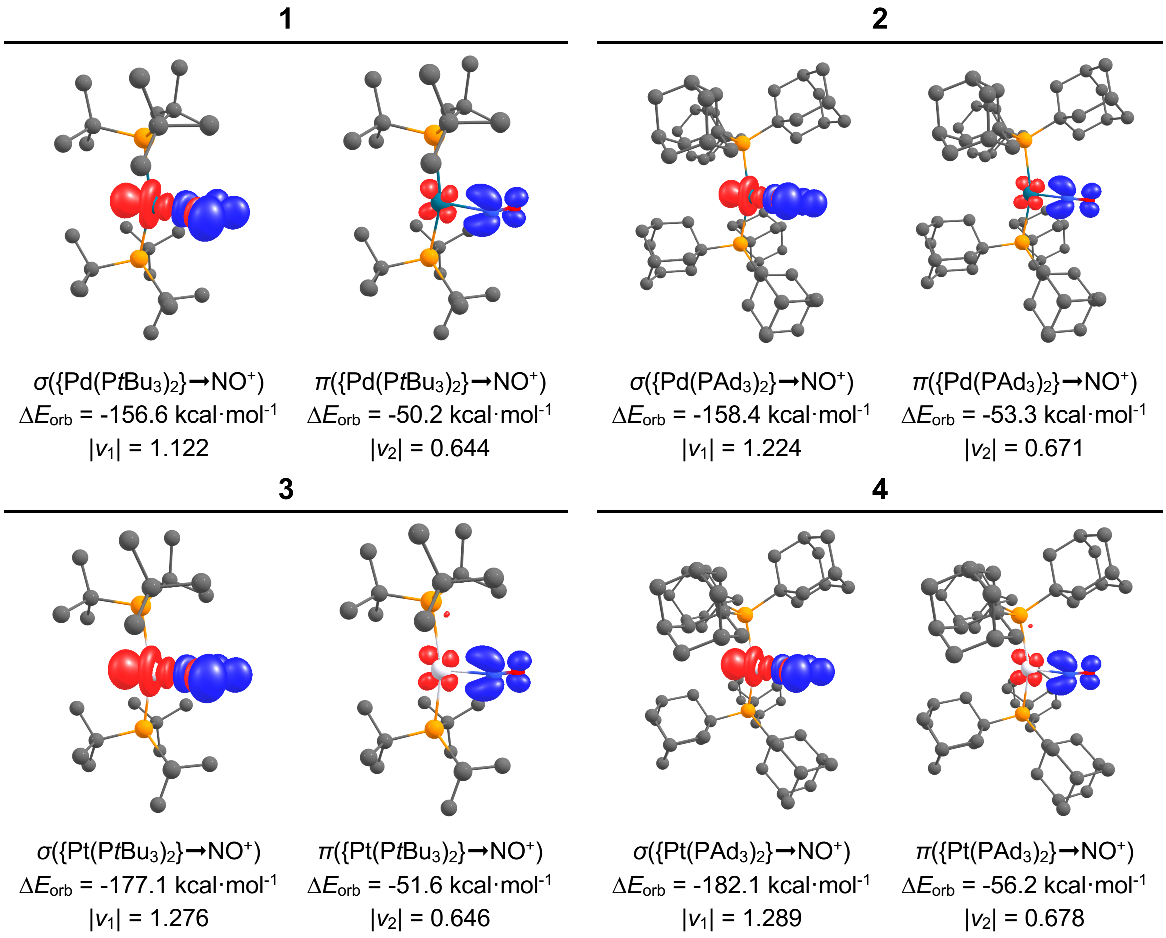

Figure S47. Leading ETS-NOCV deformation densities associated with $\{M(PR_3)_2\}/NO^+$ fragmentation of **1–4**; charge flow from red to blue. Associated energies and eigenvalues for the most important NOCV pairs.

2.4. Relaxed potential energy scans

Relaxed potential energy scans (rPESs) were carried out using Gaussian16 at the M06/def2-SVP level of theory. Variation of the ∠M–N–O angle was investigated for **1** and **3** from 121º to 176º in 5º steps, resulting in 12 conformations (coordinates provided in XYZ format). Despite repeated attempts, we were unable to optimise conformations with ideal linear nitrosyl geometries, which invariably did not converge during the optimization. In both systems, transition from a bent to linear nitrosyl coordination mode is correlated with an increase in the electronic energy, decrease in the ∠P–M–P angle, and ultimately convergence to a pseudo trigonal planar metal geometry (Figure S48).

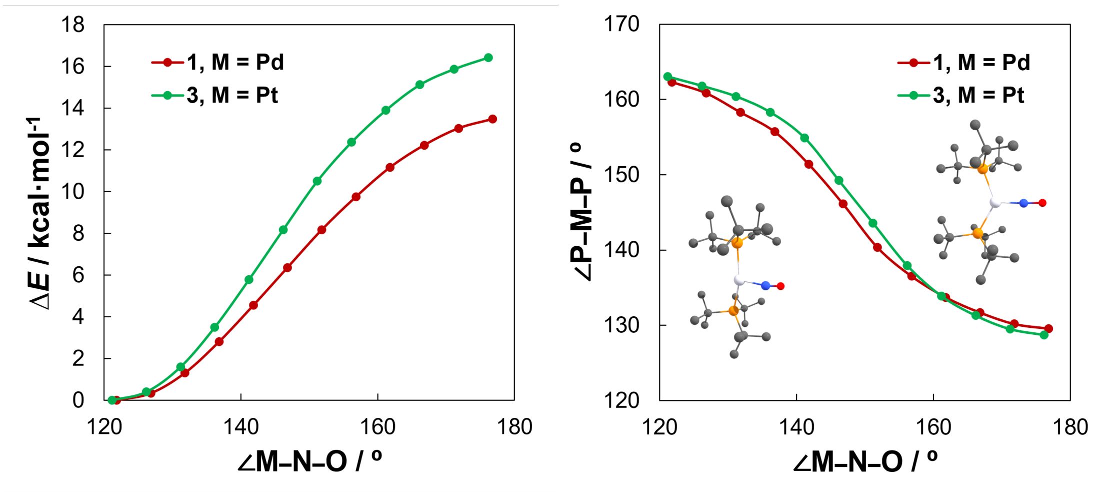

Figure S48. Relaxed potential scan for variation of the ∠M–N–O angle in **1** (red) and **3** (green). Changes in the electronic energy at the ZORA-M06/TZ2P//M06/def2-SVP level of theory (left) and ∠P–M–P bite (right) of the complexes. The conformational change of **3** along the reaction coordinate is also provided.

2.5. Activation strain model analysis

The rPESs for ∠M–N–O bending in **1** and **3** were studied using the activation strain model (ASM).[21] Briefly, the ASM involves deconvolution of the potential energy surface of a molecular system $\Delta E(\zeta)$ into two chemically meaningful terms: the total strain energy $\Delta E_{\text{strain}}(\zeta)$, which is associated with the distortion of the fragments from their equilibrium geometry and is thus related to the structural rigidity of the fragments, and the interaction energy $\Delta E_{\text{int}}(\zeta)$ between the fragments. $\Delta E_{\text{strain}}(\zeta)$ can be further deconvoluted in the contributions from each fragment, whilst $\Delta E_{\text{int}}(\zeta)$ can be deconvoluted using the EDA method as overviewed in section 2.3.

$$\Delta E(\zeta) = \Delta E_{\text{strain}}(\zeta) + \Delta E_{\text{int}}(\zeta) \qquad \textbf{Eq. S7}$$

$$\Delta E_{\text{strain}}(\zeta) = \Delta E_{\text{strain\_frag\_A}}(\zeta) + \Delta E_{\text{strain\_frag\_B}}(\zeta) \qquad \textbf{Eq. S8}$$

$$\Delta E_{\text{int}}(\zeta) = \Delta V_{\text{elstat}}(\zeta) + \Delta E_{\text{Pauli}}(\zeta) + \Delta E_{\text{orb}}(\zeta) \qquad \textbf{Eq. S9}$$

ASM analysis of all the structures generated in the rPESs was carried out using the $\{M(PtBu_3)_2\}/NO^+$ fragmentation of the metal–nitrosyl bond at the ZORA-M06/TZ2P level of theory using the open-source PyFrag module version 2019 and ADF (Table S4).[22]

Table S4. ASM analysis of ∠M–N–O bending in **1** and **3** (relative energies in kcal·mol^{-1}).

Coord. / °	∠PMP / °	ΔE	ΔE_{strain}	$\Delta E_{strain\_M(PR_3)_2}$	$\Delta E_{strain\_NO^+}$	ΔE_{int}	ΔV_{elstat}	ΔE_{Pauli}	ΔE_{orb}
rPES of the ∠Pd–N–O angle in **1**									
121.8	162.3	0.0	0.0	0.0	0.0	0.0	0.0	0.0	0.0
126.8	160.8	0.3	-0.2	0.3	-0.5	0.5	-0.6	1.8	-0.7
131.8	158.3	1.3	-0.1	0.7	-0.9	1.4	-1.0	3.4	-0.9
136.8	155.7	2.8	0.1	1.4	-1.3	2.7	-1.0	3.8	-0.1
141.8	151.4	4.5	1.0	2.9	-1.8	3.5	0.3	2.3	1.0
146.8	146.1	6.4	2.9	5.1	-2.3	3.5	2.4	-0.7	1.9
151.8	140.4	8.2	5.6	8.2	-2.7	2.6	5.6	-6.0	3.0
156.8	136.5	9.7	7.6	10.5	-2.9	2.1	8.5	-11.1	4.8
161.8	133.7	11.2	9.3	12.4	-3.1	1.9	11.0	-15.7	6.5
166.8	131.7	12.2	10.6	13.8	-3.2	1.6	13.2	-19.8	8.2
171.8	130.2	13.0	11.6	14.9	-3.3	1.4	14.8	-23.0	9.5
176.8	129.6	13.5	12.0	15.4	-3.4	1.4	15.6	-24.4	10.2
rPES of the ∠Pt–N–O angle in **3**									
121.2	163.0	0.0	0.0	0.0	0.0	0.0	0.0	0.0	0.0
126.2	161.8	0.4	-0.1	0.4	-0.5	0.5	-1.0	3.0	-1.4
131.2	160.4	1.6	-0.2	0.7	-1.0	1.8	-2.2	5.8	-1.8
136.2	158.3	3.5	0.0	1.5	-1.4	3.4	-2.9	7.6	-1.3
141.2	154.9	5.8	1.1	3.0	-1.9	4.7	-3.3	8.7	-0.8
146.2	149.3	8.2	3.9	6.1	-2.3	4.3	-3.5	9.8	-1.9
151.2	143.6	10.5	8.1	10.7	-2.6	2.4	-2.4	8.3	-3.5
156.2	137.9	12.4	13.3	16.2	-2.9	-0.9	-0.1	4.4	-5.2
161.2	133.9	13.9	17.5	20.6	-3.1	-3.6	2.1	0.3	-6.1
166.2	131.3	15.1	20.4	23.7	-3.3	-5.3	4.1	-3.7	-5.8
171.2	129.5	15.9	22.5	25.9	-3.4	-6.6	5.8	-7.1	-5.4
176.2	128.7	16.4	23.5	26.9	-3.4	-7.1	6.6	-8.6	-5.1

2.6. Analysis of the ∠M–N–O rPESs

Transition from a bent to a linear nitrosyl coordination mode over the scans is associated with a considerable energetic penalty (13.5 kcal·mol^{-1} for **1** and 16.4 kcal·mol^{-1} for **3**) and ASM analysis identifies ΔE_{strain} as the origin of this destabilisation. In the case of **1**, ΔE_{int} increases 3.5 kcal·mol^{-1} during the first half of the scan (121° to 146°) before decreasing and plateauing at +1.4 kcal·mol^{-1}

(Figure S49A). When favourable $\Delta E_{strain\_NO^+}$ is factored in, the metal-nitrosyl interaction is found to be stabilised by 1.9 kcal·mol^{-1} at the end of the scan. This is not enough to offset the considerable energetic penalty associated with distortion of the {Pd(P*t*Bu$_3$)$_2$} fragment over the scan, which is associated with an uninterrupted increase in $\Delta E_{strain\_Pd(PtBu_3)_2}$ up to 15.4 kcal·mol^{-1} over the scan (Figure S49B). Similarly for **3**, but in this case ΔE_{strain} increases up to 23.5 kcal·mol^{-1} and ΔE_{int} ultimately plateaus at -7.1 kcal·mol^{-1} (Figure S49C). When favourable $\Delta E_{strain\_NO^+}$ is included, the metal-nitrosyl interaction is found to be stabilised by 10.5 kcal·mol^{-1} at the end of the scan. This is more pronounced than in **1**, but distortion of the {Pt(P*t*Bu$_3$)$_2$} fragment over the scan is associated with an even higher energetic penalty (26.9 kcal·mol^{-1}, Figure S49D).

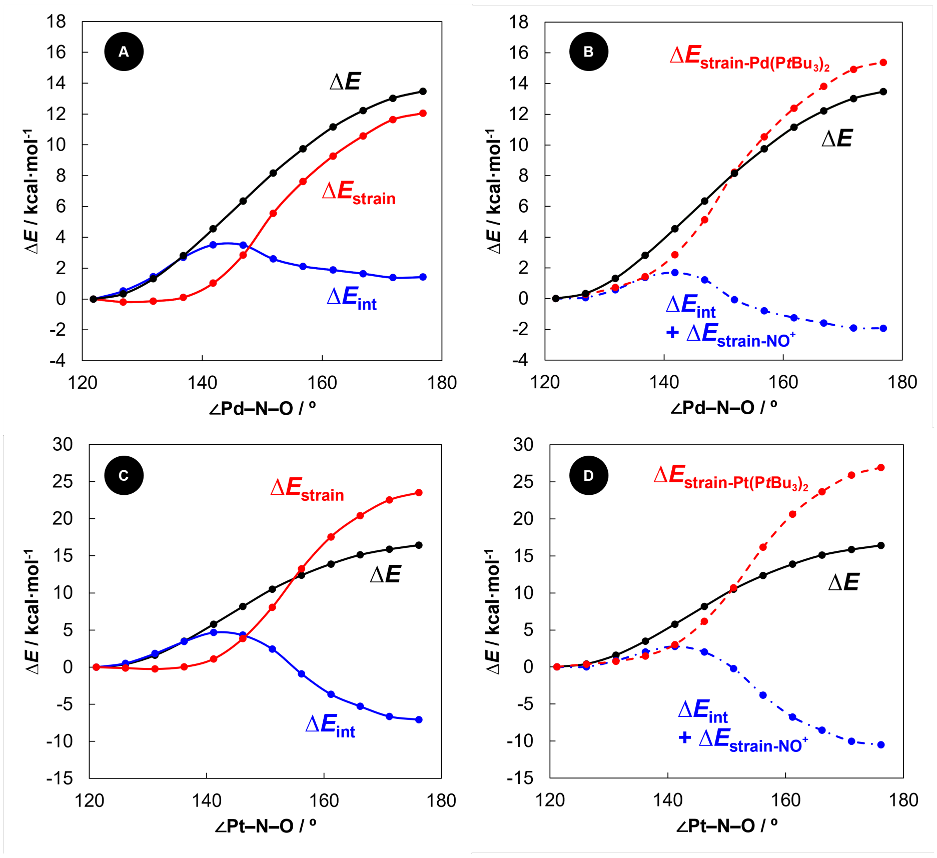

Figure S49. ASM analysis of ∠M–N–O bending in **1** (**A**, **B**) and **3** (**C**, **D**).

To help rationalise the ASM findings, we have examined the qualitative Walsh diagram for a generic linear d^{10}-ML$_2$ fragment (Figure S50)[23] and analysed the linear isomers of **1** and **3** by the EOS (Table S5 and Figure S51) and EDA (Table S6 and Figure S52) methods, as described above.

Inspection of the Walsh diagram demonstrates how the M–N–O and the P–M–P angles are electronically correlated. As the {M(P*t*Bu$_3$)$_2$} fragments are distorted away from linearity, the energy of the 2b$_1$ orbital sharply rises and becomes more π-basic. This is evident from the EOS and EDA analysis, where the leading metal-nitrosyl interaction in the linear isomers is now a polarised $d_{xz}/\pi^*_{(\parallel)}$

π-backbonding interaction. As the nitrosyl is tilted, the polarised σ-bonding $d_{Z^2}/\pi^*_{(\perp)}$ interaction becomes symmetry forbidden and is replaced by a highly polarised π-backbonding $d_{xy}/\pi^*_{(\perp)}$ interaction. The driving force for the observed correlation is therefore the electronic stabilisation provided by enhanced in-plane metal-nitrosyl π bonding. The Walsh diagram also demonstrates that distortion away from a linear ML_2 coordination geometry is inherently unfavourable and has been attributed to increasing Pauli repulsion between the ligand donor orbitals as destabilisation of the metal-ligand σ* orbitals.[23] In this case, steric repulsion between the two bulky phosphine ligands will significantly amplify the energetic penalty. It is interesting to note that there is no significant change in the extent of Pauli repulsion on adopting the linear nitrosyl coordination mode. Metal interaction with the nitrogen lone pair remains repulsive and there is no recognisable σ-bond present.

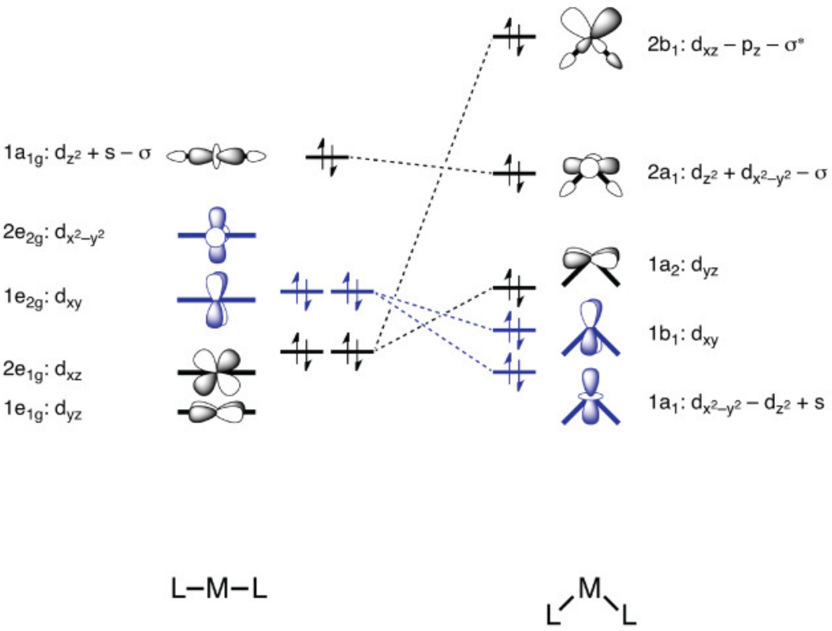

Figure S50. Qualitative Walsh diagram showing how the energy of the frontier orbitals change on bending a generic d^{10}-ML_2 fragment. Reproduced from ref. 23.

Table S5. EOS analysis of bent/linear **1** and **3** at the M06/def2-TZVP//M06/def2-SVP level of theory.

	OS		*R*(%)	EFO population					
	**M**	**NO**		d_{z^2}/d_{xy}	$\pi^*_{(\perp)}$	**%NO**	d_{xz}	$\pi^*_{(\parallel)}$	**%NO**
**1**	0	+1	76.9	0.632	0.363	36.5	0.787	0.162	17.1
**1**-176°	0	+1	79.6	0.779	0.160	17.0	0.673	0.377	35.9
**3**	0	+1	68.1	0.572	0.391	40.6	0.750	0.172	18.7
**3**-176°	0	+1	71.2	0.736	0.194	20.9	0.625	0.413	39.8

1-176º

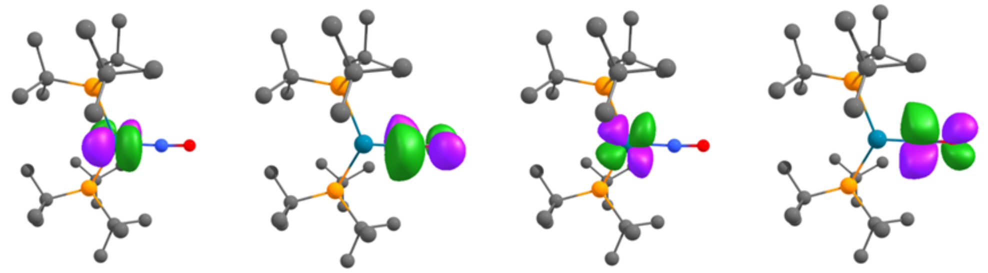

d_{xy} (0.779) $\pi^*_{(\perp)}$ (0.160) d_{xz} (0.673) $\pi^*_{(\parallel)}$ (0.377)

3-176º

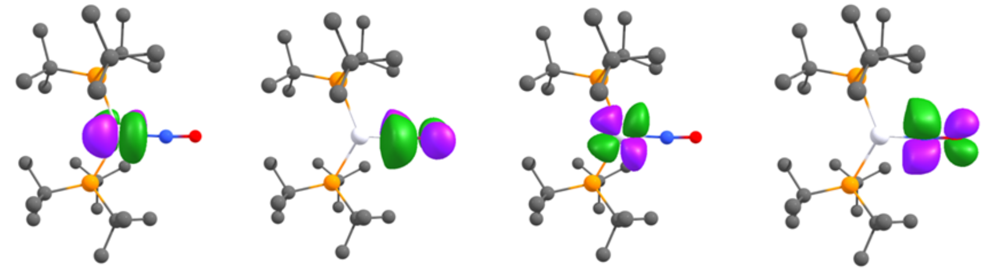

d_{xy} (0.736) $\pi^*_{(\perp)}$ (0.194) d_{xz} (0.625) $\pi^*_{(\parallel)}$ (0.413)

Figure S51. EFOs relevant to the M–NO bonds in linear **1** and **3**, with occupancies in parentheses.

Table S6. EDA-NOCV analysis for {M(P*t*Bu_3)$_2$}/NO^+ fragmentation in bent/linear **1** and **3** (energies in kcal·mol^{-1}).

	**1**	**1**-176º	**3**	**3**-176º
ΔE_{int}	-130.7	-129.3	-139.8	-147.2
ΔE_{Pauli}	+206.7	+182.4	+253.4	+251.4
ΔV_{elstat}	-114.2	-98.6	-135.5	-131.8
ΔE_{orb}	-223.2	-213.1	-257.7	-266.8
ΔE_{σ} (%)	-156.6 (64.4)	-	-177.1 (65.1)	-
ΔE_{π} (%)	-50.2 (20.6)	-178.9 (76.0)	-51.6 (19.0)	-206.5 (72.5)
ΔE_{rest} (%)	-36.6 (15.0)	-56.5 (24.0)	-43.4 (15.9)	-78.3 (27.5)
ΔE_{prep}	+22.5	+34.6	+26.3	+50.1
ΔE_{bind} (= $-D_e$)	-108.2	-94.7	-113.5	-97.1

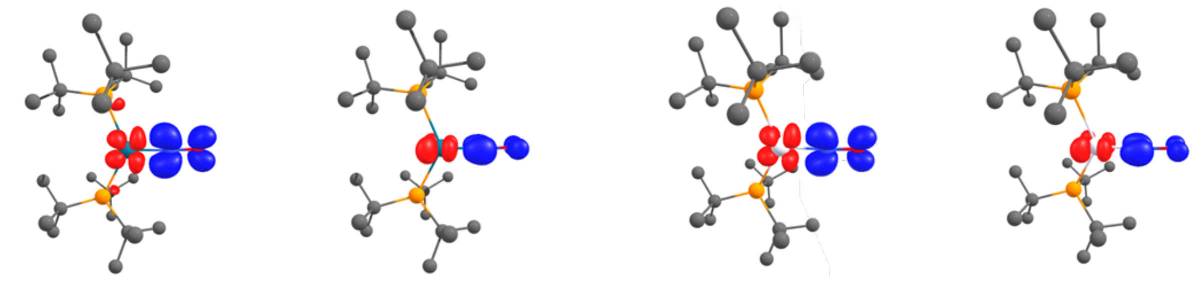

Figure S52. Leading ETS-NOCV deformation densities associated with $\{M(PtBu_3)_2\}/NO^+$ fragmentation of bent **1** and **3**; charge flow from red to blue. Associated energies and eigenvalues for the most important NOCV pairs.

3. References

1 Pike, S. D.; Crimmin, M. R.; Chaplin, A. B. Organometallic Chemistry Using Partially Fluorinated Benzenes. *Chem. Commun.* **2017**, *53*, 3615–3633.

2 Krämer, T.; Gyton, M. R.; Bustos, I.; Sinclair, M. J. G.; Tan, S.; Wedge, C. J.; Macgregor, S. A.; Chaplin, A. B. Stability and C–H Bond Activation Reactions of Palladium(I) and Platinum(I) Metalloradicals: Carbon-to-Metal H-Atom Transfer and an Organometallic Radical Rebound Mechanism. *J. Am. Chem. Soc.* **2023**, *145*, 14087–14100.

3 Brauer, G. *Handbook of Preparative Inorganic Chemistry* (2nd Ed.), Academic Press, **1963**, Vol. 1, pp 485–487.

4 (a) Martínez-Martínez, A. J.; Weller, A. S. Solvent-Free Anhydrous Li^+, Na^+ and K^+ Salts of $[B(3,5\text{-}(CF_3)_2C_6H_3)_4]^-$, $[BAr^F{}_4]^-$. Improved Synthesis and Solid-State Structures. *Dalton Trans.* **2019**, *48*, 3551–3554; (b) Buschman, W. E.; Miller, J. S.; Bowman-James, K.; Miller, C. N. Synthesis of $[M^{II}(NCMe)_6]^{2+}$ (M= V, Cr, Mn, Fe, Co, Ni) Salts of Tetra [3,5-bis(trifluoromethyl)phenyl] Borate. *Inorg. Synth.* **2002**, *33*, 83–91.

5 Pregosin, P. S. *NMR in Organometallic Chemistry*, Wiley, **2012**, pp. 251–254.

6 Martin, G. E.; Hadden, C. E. Long-Range ^1H–^{15}N Heteronuclear Shift Correlation at Natural Abundance. *J. Nat. Prod.* **2000**, *63*, 543–585.

7 Frisch, M. J.; Trucks, G. W.; Schlegel, H. B.; Scuseria, G. E.; Robb, M. A.; Cheeseman, J. R.; Scalmani, G.; Barone, V.; Petersson, G. A.; Nakatsuji, H.; Li, X.; Caricato, M.; Marenich, A. V.; Bloino, J.; Janesko, B. G.; Gomperts, R.; Mennucci, B.; Hratchian, H. P.; Ortiz, J. V.; Izmaylov,

A. F.; Sonnenberg, J. L.; Williams-Young, D.; Ding, F.; Lipparini, F.; Egidi, F.; Goings, J.; Peng, B.; Petrone, A.; Henderson, T.; Ranasinghe, D.; Zakrzewski, V. G.; Gao, J.; Rega, N.; Zheng, G.; Liang, W.; Hada, M.; Ehara, M.; Toyota, K.; Fukuda, R.; Hasegawa, J.; Ishida, M.; Nakajima, T.; Honda, Y.; Kitao, O.; Nakai, H.; Vreven, T.; Throssell, K.; Montgomery, J. A., Jr.; Peralta, J. E.; Ogliaro, F.; Bearpark, M. J.; Heyd, J. J.; Brothers, E. N.; Kudin, K. N.; Staroverov, V. N.; Keith, T. A.; Kobayashi, R.; Normand, J.; Raghavachari, K.; Rendell, A. P.; Burant, J. C.; Iyengar, S. S.; Tomasi, J.; Cossi, M.; Millam, J. M.; Klene, M.; Adamo, C.; Cammi, R.; Ochterski, J. W.; Martin, R. L.; Morokuma, K.; Farkas, O.; Foresman, J. B.; Fox, D. J. *Gaussian 16*, Revision A.03; Gaussian, Inc.: Wallingford CT, **2016**.

[8] Zhao, Y.; Truhlar, D. G. A New Local Density Functional for Main-Group Thermochemistry, Transition Metal Bonding, Thermochemical Kinetics, and Noncovalent Interactions. *J. Chem. Phys.* **2006**, *125*, 194101.

[9] (a) Erigend, F. Accurate Coulomb-Fitting Basis Sets for H to Rn. *Phys. Chem. Chem. Phys.* **2006**, *8*, 1057–1065; (b) Weigend, F.; Ahlrichs, R. Balanced Basis Sets of Split Valence, Triple Zeta Valence and Quadruple Zeta Valence Quality for H to Rn: Design and Assessment of Accuracy. *Phys. Chem. Chem. Phys.* **2005**, *7*, 3297–3305.

[10] (a) Postils, V.; Delgado-Alonso, C.; Luis, J. M.; Salvador, P. An Objective Alternative to IUPAC's Approach to Assign Oxidation States. *Angew. Chem. Int. Ed.* **2018**, *57*, 10525–10529; (b) Ramos-Cordoba, E.; Postils, V.; Salvador, P. Oxidation States from Wave Function Analysis. *J. Chem. Theory Comput.* **2015**, *11*, 1501–1508.

[11] Salvador, P.; Ramos-Cordoba, E.; Gimferrer, M.; Montilla, M. *Program APOST-3D*, Version 4, Girona, **2020**.

[12] Salvador, P.; Ramos-Cordoba, E. An Approximation to Bader's Topological Atom. *J. Chem. Phys.* **2013**, **139**, 071103–071104.

[13] (a) Becke*,* A. D. Density-Functional Exchange-Energy Approximation with Correct Asymptotic Behavior. *Phys. Rev. A* **1988**, *38* 3098–3100; (b) Perdew, J. P. Density-Functional Approximation for the Correlation Energy of the Inhomogeneous Electron. Gas *Phys. Rev. B* **1986**, *33*, 8822–8824; (c) Grimme, S.; Antony, J.; Ehrlich, S.; Krieg, H. A Consistend and Accurate *Ab Initio* Parametrization of Density Functional Dispersion Correction (DFT-D) for the 94 Elements H-Pu. *J. Chem. Phys.* **2010**, *132*, 154104; (d) Grimme, S.; Ehrlich, S.; Goerigk, L. Effect of the Damping Function in Dispersion Corrected Density Functional Theory. *J. Comput. Chem.* **2011**, *32*, 1456-1465.

[14] Schröder, D.; Klüfers, P. Bonding in PdCl(NO) and Related Nitrosylmetal Species of the Enemark–Feltham $\{MNO\}^{10}$ Type. *Z. für Anorg. Allg. Chem.* **2023**, *649*, e202200338.

[15] Ampßler, T.; Monsch, G.; Popp, J.; Riggenmann, T.; Salvador, P.; Schröder, D.; Klüfers, P. Not Guilty on Every Count: The "Non-Innocent" Nitrosyl Ligand in the Framework of IUPAC2s Oxidation-State Formalism. *Angew. Chem. Int. Ed.* **2020**, *59*, 12381–12386.

[16] (a) Bickelhaupt, F. M.; Baerends, E. J. Kohn-Sham Density Functional Theory: Predicting and Understanding Chemistry. *Rev. Comput. Chem.* **2000**, *15*, 1–86. (b) Ziegler, T.; Rauk, A. On the Calculation of Bonding Energies by the Hartree Fock Slater Method. *Theor. Chim. Acta* **1977**, *46*, 1–10; (c) Morokuma, K. Molecular Orbital Studies of Hydrogen Bonds. III. C=O···H–O Hydrogen Bond in $H_2CO\cdots H_2O$ and $H_2CO\cdots 2H_2O$. *J. Chem. Phys.* **1971**, *55*, 1236–1244.

[17] (a) Zhao, L.; von Hopffgarten, M.; Andrada, D. M.; Frenking, G. Energy Decomposition Analysis. *WIREs Comput. Mol. Sci.* **2018**, *8,* e1345; (b) Mitoraj, M. P.; Michalak, A.; Ziegler, T. A Combined Charge and Energy Decomposition Scheme for Bond Analysis. *J. Chem. Theory Comput.* **2009**, *5*, 962–975.

[18] AMS2020.102, SCM, Theoretical Chemistry, Vrije Universiteit, Amsterdam, The Netherlands, http://www.scm.com.

[19] Van Lenthe, E.; Baerends, E. J. Optimized Slater-Type Basis Sets for the Elements 1-118. *J. Comput. Chem.* **2003**, *24*, 1142–1156.

[20] (a) Van Lenthe, E.; Baerends, E. J.; Snijders, J. G. Relativistic Total Energy Using Regular Approximations. *J. Chem. Phys.* **1994**, *101*, 9783–9792; (b) Van Lenthe, E.; Baerends, E. J.; Snijders, J. G. Relativistic Regular Two-Component Hamiltonians. *J. Chem. Phys.* **1993**, *99*, 4597–4610.

[21] Vermeeren, P.; van der Lubbe, S.C.C.; Fonseca Guerra, C.; Bickelhaupt, F. M.; Hamlin, T. A. Understanding Chemical Reactivity Using the Activation Strain Model. *Nat. Protoc*. **2020**, *15*, 649–667.

[22] (a) Sun, X.; Soini, T. M.; Poater, J.; Hamlin, T. A.; Bickelhaupt, F. M. PyFrag 2019 – Automating the Exploration and Analysis of Reaction Mechanisms. *J. Comput. Chem.* **2019**, *40*, 2227–2233; (b) te Velde, G.; Bickelhaupt, F. M.; Baerends, E. J.; Fonseca Guerra, C.; van Gisbergen, S. J. A.; Snijders, J. G.; Ziegler, T. Chemistry with ADF. *J. Comput. Chem.* **2001**, *22*, 931–967.

[23] (a) Wolters, L. P.; Bickelhaupt, F. M. The Activation Strain Model and Molecular Orbital Theory. *WIREs Comput. Mol. Sci.* **2015**, *5*, 324–343; (b) Wolters, L. P.; Bickelhaupt, F. M. Nonlinear d^{10}-ML_2 Transition-Metal Complexes. *ChemistryOpen* **2013**, *2*, 106–114.
